# Functional conservation of the apoptotic machinery from coral to man: the diverse and complex Bcl-2 and caspase repertoires of *Acropora millepora*

**DOI:** 10.1186/s12864-015-2355-x

**Published:** 2016-01-16

**Authors:** Aurelie Moya, Kazuhiro Sakamaki, Benjamin M. Mason, Lotte Huisman, Sylvain Forêt, Yvonne Weiss, Tara E. Bull, Kentaro Tomii, Kenichiro Imai, David C. Hayward, Eldon E. Ball, David J. Miller

**Affiliations:** ARC Centre of Excellence for Coral Reef Studies, James Cook University, Townsville, Queensland 4811 Australia; Department of Animal Development and Physiology, Graduate School of Biostudies, Kyoto University, Kyoto, 606-8501 Japan; Comparative Genomics Centre and Department of Molecular and Cell Biology, James Cook University, Townsville, Queensland 4811 Australia; Section of Computational Science, Universiteit van Amsterdam, Science Park 904, 1098 XH Amsterdam, The Netherlands; Evolution, Ecology and Genetics, Research School of Biology, Australian National University, Bldg. 46, Canberra, ACT 0200 Australia; Biotechnology Research Institute for Drug Discovery, Department of Life Science and Biotechnology, National Institute of Advanced Industrial Science and Technology (AIST), Tokyo, 135-0064 Japan

**Keywords:** Apoptosis, Caspase, Bcl-2, Coral, *Acropora millepora*, Cnidaria

## Abstract

**Background:**

Apoptotic cell death is a defining and ubiquitous characteristic of metazoans, but its evolutionary origins are unclear. Although *Caenorhabditis* and *Drosophila* played key roles in establishing the molecular bases of apoptosis, it is now clear that cell death pathways of these animals do not reflect ancestral characteristics. Conversely, recent work suggests that the apoptotic networks of cnidarians may be complex and vertebrate-like, hence characterization of the apoptotic complement of representatives of the basal cnidarian class Anthozoa will help us to understand the evolution of the vertebrate apoptotic network.

**Results:**

We describe the Bcl-2 and caspase protein repertoires of the coral *Acropora millepora*, making use of the comprehensive transcriptomic data available for this species. Molecular phylogenetics indicates that some *Acropora* proteins are orthologs of specific mammalian pro-apoptotic Bcl-2 family members, but the relationships of other Bcl-2 and caspases are unclear. The pro- or anti-apoptotic activities of coral Bcl-2 proteins were investigated by expression in mammalian cells, and the results imply functional conservation of the effector/anti-apoptotic machinery despite limited sequence conservation in the anti-apoptotic Bcl-2 proteins. A novel caspase type (“Caspase-X”), containing both inactive and active caspase domains, was identified in *Acropora* and appears to be restricted to corals. When expressed in mammalian cells, full-length caspase-X caused loss of viability, and a truncated version containing only the active domain was more effective in inducing cell death, suggesting that the inactive domain might modulate activity in the full-length protein. Structure prediction suggests that the active and inactive caspase domains in caspase-X are likely to interact, resulting in a structure resembling that of the active domain in procaspase-8 and the inactive caspase domain in the mammalian c-FLIP anti-apoptotic factor.

**Conclusions:**

The data presented here confirm that many of the basic mechanisms involved in both the intrinsic and extrinsic apoptotic pathways were in place in the common ancestor of cnidarians and bilaterians. With the identification of most or all of the repertoires of coral Bcl-2 and caspases, our results not only provide new perspectives on the evolution of apoptotic pathways, but also a framework for future experimental studies towards a complete understanding of coral bleaching mechanisms, in which apoptotic cell death might be involved.

**Electronic supplementary material:**

The online version of this article (doi:10.1186/s12864-015-2355-x) contains supplementary material, which is available to authorized users.

## Background

**Fig. 1:**
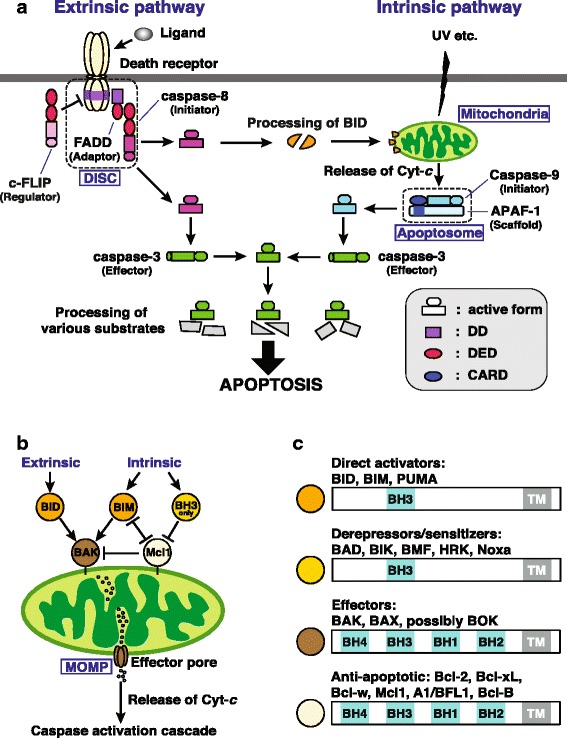
The involvement of caspases and Bcl-2 family proteins in the apoptotic pathways of mammals. **a** Apoptosis can be triggered either by extrinsic ligands binding to death receptors on the cell surface, or by intrinsic stimuli acting at the level of mitochondrial membrane integrity. These pathways enable the activation of caspases, a class of cysteine aspartyl proteases, and these bring about the orderly destruction of the cell. The present study indicates that the coral apoptotic system is vertebrate-like, comprising components of both extrinsic and intrinsic cell death pathways (see Discussion section). **b** The mammalian Bcl-2 (B cell lymphoma-2) protein family contains both pro- and anti-apoptotic members. Multi-domain Bcl-2 ‘effectors’ (in brown on the figure) mediate mitochondrial outer-membrane permeabilization (MOMP) while the ‘anti-apoptotic’ multi-domain family members (shown in cream) inhibit it. BH3-only proteins share only the third Bcl Homology (BH) domain with other members of the Bcl-2 family, and regulate the other two classes either by directly activating the pro-apoptotic effectors (‘direct activators’; shown in orange) or by disrupting existing anti-apoptotic complexes without directly causing MOMP (‘derepressors or sensitizers’; shown in yellow). **c** Structure of the mammalian candidates for the direct activators (BID, Bcl-2 interacting domain death antagonist; BIM, Bcl-2 interacting mediator of cell death; PUMA, p53-upregulated modulator of apoptosis), the derepressors/sensitizers (BAD, Bcl-2 antagonist of cell death; BIK, Bcl-2 interacting killer; BMF, Bcl-2 modifying factor; HRK, Harakiri; Noxa), the effectors (BAK, Bcl-2 antagonist killer 1; BAX, Bcl-2-associated X protein; BOK, Bcl-2-related ovarian killer) and the anti-apoptotic (Bcl-2, Bcl-xL, Bcl-w, Mcl1, myeloid cell leukemia 1; A1, Bcl-2-related gene A1, Bcl-B) Bcl-2 components. **b** and **c** are modified from Moldoveanu et al. [53]. Abbreviations: APAF-1, apoptotic peptidase-activating factor 1; caspase, cysteine-dependent aspartyl-specific protease; CARD, caspase recruitment domain; Cyt-*c*, Cytochrome *c*; c-FLIP, cellular FLICE-like inhibitory protein; DD, death domain; DED, death effector domain; FADD, Fas-associated death domain protein; TM, transmembrane

The apoptotic pathway of programmed cell death, which was discovered in *Caenorhabditis elegans,* is thought to be an ancient metazoan innovation on the basis of the apparent conservation of some key components from sponges to mammals [1, 2]. Apoptosis not only serves to minimize collateral damage following stress or cellular insult, but also plays critical roles in development, morphogenesis and immunity. Jacobson et al. [3] noted four functions of apoptosis: **sculpting**, as in removal of the webbing between digits in the developing mammal; **deleting structures,** as in removal of the tail of the developing frog; **adjusting cell numbers,** as in the nervous systems of both vertebrates and invertebrates and **eliminating dangerous or injured cells**, as in the elimination of defective T and B lymphocytes in the vertebrate immune system. Apoptosis has been most extensively studied in mammals, where it can be triggered either by extrinsic ligands binding to death receptors on the cell surface, or by intrinsic stimuli acting at the level of mitochondrial membrane integrity (Fig. 1a). These pathways enable the activation of caspases, a class of cysteine aspartyl proteases, and these bring about the orderly destruction of the cell.

In mammals, a suite of caspases is present, members of which have a variety of specialized roles that are to some extent reflected in domain structure. The mammalian caspases are classified into three groups based on their domain architecture: (1) those that contain only the catalytic domain (i.e. peptidase_C14) are generally downstream effector caspases, such as caspases−3, −6, −7 and −14; (2) those that also contain a tandem pair of Death Effector Domains (DED) are initiator caspases, such as caspases-8 and −10; and (3) those that also contain a Caspase Activation and Recruitment Domain (CARD) are either initiator caspases involved in the intrinsic pathway, (caspases-2 (Golgi) and −9 (mitochondria)), or those involved in activating pro-inflammatory cytokines; caspases−1, −4 −5, −11, and −12 [4].

Members of the Bcl-2 protein family are key regulators of apoptotic cell death in mammals; some prevent apoptosis, whereas others are pro-apoptotic (Fig. 1b, c). Although the older literature suggested that the anti- and pro- apoptotic Bcl-2 proteins could be distinguished on the basis of Bcl-2 Homology (BH) domain architecture [5], this view is no longer tenable, as it is now recognized that several pro-apoptotic proteins share with their anti-apoptotic counterparts the presence of 4 BH domains and a similar overall (globular) structure [6]. Bcl-2 family members have a variety of modes of action. During apoptosis, conformational changes in the pro-apoptotic proteins Bax (Bcl-2-Associated X protein) and Bak (Bcl-2 Antagonist Killer 1) generate oligomeric forms that are capable of making the outer mitochondrial membrane permeable, resulting in release of cytochrome *c* (Cyt-*c*), which binds to the scaffold protein APAF-1 (Apoptotic Peptidase-Activating Factor 1) and ultimately activates effector caspases (e.g., caspase-3, Fig. 1a, b). Many anti-apoptotic Bcl-2 proteins act by binding to activated Bax or Bak, and preventing their oligomerisation; alternatively anti-apoptotic activity may be imposed by Bcl-2 proteins sequestering the ligands that drive activation of Bax or Bak [6].

There are some similarities between the apoptotic systems of mammals and those of the model invertebrates *Drosophila* and *Caenorhabditis*, but the extrinsic apoptotic pathway is not present in either of these latter, and the intrinsic pathways differ in some critical respects and are considerably less complex. Rather than controlling the release of cytochrome *c* from mitochondria, in both organisms Bcl-2 proteins are thought to act primarily at the level of the apoptosome. *Caenorhabditis* Ced-9, the sole multi-domain Bcl-2 protein present, functions by sequestering the APAF-1 homolog (Ced-4), thus inhibiting apoptosis. Buffy, one of the two Bcl-2 family members in *Drosophila*, is thought to act in a similar manner, the pro-apoptotic Bcl-2 protein Debcl potentially relieving this inhibition by dimerising with Buffy [7]. Caspase-8, the key player in the extrinsic cell-death pathway, is an evolutionarily ancient molecule [8], but has been lost in the case of *Caenorhabiditis*, as have death receptors and FADD (Fas-associated protein with Death Domain). Surveys of the available whole genome sequences suggest that the simplicity of the apoptotic networks of *Drosophila* and *Caenorhabditis* is unlikely to reflect the ancestral condition. It is unclear, however, how far back in evolution control of mitochondrial permeability by Bcl-2 proteins was in place.

Apoptosis has remained less well studied in other invertebrates, although studies on the hydrozoan cnidarians *Hydra magnipapillata* (summarized in [9, 10]) and *Hydractinia echinata* (e.g., [11–13]) indicate the presence of complex repertoires of apoptotic proteins. Corals (and sea anemones as surrogate corals) have been a major focus of attention due to interest in the possible roles of apoptosis in coral “bleaching”.

A common response of corals to many different stresses, or combinations of stresses, is to bleach, where bleaching is defined as the loss of their endosymbiotic *Symbiodinium* or the loss by them of their photosynthetic pigments. The most widespread cause of bleaching recognized at present is overheating, as a result of global warming. A widely accepted theory is that bleaching is due to reactive oxygen species generated by photo-oxidative damage [14], but while that may be a partial explanation, it is not the whole story, as corals also bleach when heated in the dark [15]. If photooxidative damage were indeed the main cause of apoptosis then *Symbiodinium* loss could be a means of reducing the degree of damage, but presumably if the damage was too severe then the caspase cascade would continue. Tchernov et al. [16] explain the delay between loss of symbionts and cell death by speculating that after the loss of symbionts ROS levels continue to rise as a result of mitochondrial dysfunction. Kvitt et al. [17] carried out detailed cell biological and molecular studies that led them to the following scenario for corals that ultimately survive thermal stress. Firstly there was an initial onset of apoptosis which was accompanied by activation of anti-oxidant/anti-apoptotic mediators that blocked progression of apoptosis to other cells. This was followed by “acclimatization of the coral to the chronic thermal stress alongside the completion of symbiosis breakdown”. Pernice et al. [18] proposed a similar progression, with an initial burst of caspase-mediated apoptosis, followed by a delayed Bcl-mediated protective response in response to heat stress.

Despite the recent interest in apoptosis in corals, few studies have focused specifically on the apoptotic machinery of anthozoans. Dunn et al. [19] discussed the genes involved in apoptosis in the sea anemone, *Aiptasia*, and a preliminary survey of apoptotic components of *Nematostella vectensis* has been carried out [20].

Characterization of the apoptotic machinery of corals is important because it can not only provide novel evolutionary perspectives, but also lead to a much better understanding of the roles of apoptosis in coral bleaching and stress responses. Because reef-building corals are difficult to maintain in aquaria, to date many of the studies aimed at understanding coral cell death as a result of bleaching have instead used the sea anemones *Aiptasia* or *Anemonia* as surrogates; not only are these hardy and simple to culture, but they also host the same genus of photosynthetic symbionts as corals. In one of the few papers to directly address coral apoptosis at a molecular level, Tchernov et al. [16] demonstrated that the pan-caspase inhibitor zVAD-FMK inhibited heat induced apoptosis and bleaching in *Pocillopora damicornis* and *Montipora capitata*. A number of Bcl-2, caspase or other apoptosis-related genes have been identified in corals using candidate gene (e.g., [17, 18, 21]), microarray (e.g., [22–24]), transcriptomic (e.g., [25]) or genomic [26] approaches. However, studies to date have been piecemeal rather than comprehensive. Despite the lack of complete surveys of caspase or Bcl-2 repertoires, it is clear that the apoptotic pathways of corals are more complex than are those of the model invertebrates *Caenorhabditis* and *Drosophila*, and are probably more like those of vertebrates. TNFα-induced apoptosis has recently been reported in *Acropora* [27], unequivocally demonstrating the operation of the extrinsic cell death pathway, and the *Acropora* caspase-8 and FADD proteins have been shown to interact [8], suggesting some fundamental similarities between the apoptotic networks of corals and mammals. Caspases are also present in corals, but of these only the caspase-8 ortholog has been characterized [8]. A number of Bcl-2 family members, nominally identified as corresponding to Bcl-2 (HO088736; [23]), and the pro-apoptotic proteins Bax (DY581529; [21]) and Bak (EZ037140.1; [18]) have previously been reported from *Acropora*. However, until now there has not been a systematic study of the Bcl-2 complement of any coral and no functional data are available for any of the coral Bcl-2 family members.

In a recent comparison of acute (3d) and longer-term (9d) responses of the coral *Acropora millepora* to CO_2_-induced ocean acidification [25], we found that a suite of Bcl-2 genes was up-regulated in the longer-term exposure. Our efforts to understand the functional significance of this response led to the present study, in which we characterize the multi-domain Bcl-2 and caspase gene repertoires of *A. millepora*, and place these in an evolutionary context. With the identification of most or all of the repertoires of coral Bcl-2 and caspases, our results not only provide new perspectives on the evolution of apoptotic pathways, but also a framework for future experimental studies towards a complete understanding of coral bleaching mechanisms.

## Results

### The *Acropora* Bcl-2 repertoire

**Fig. 2:**
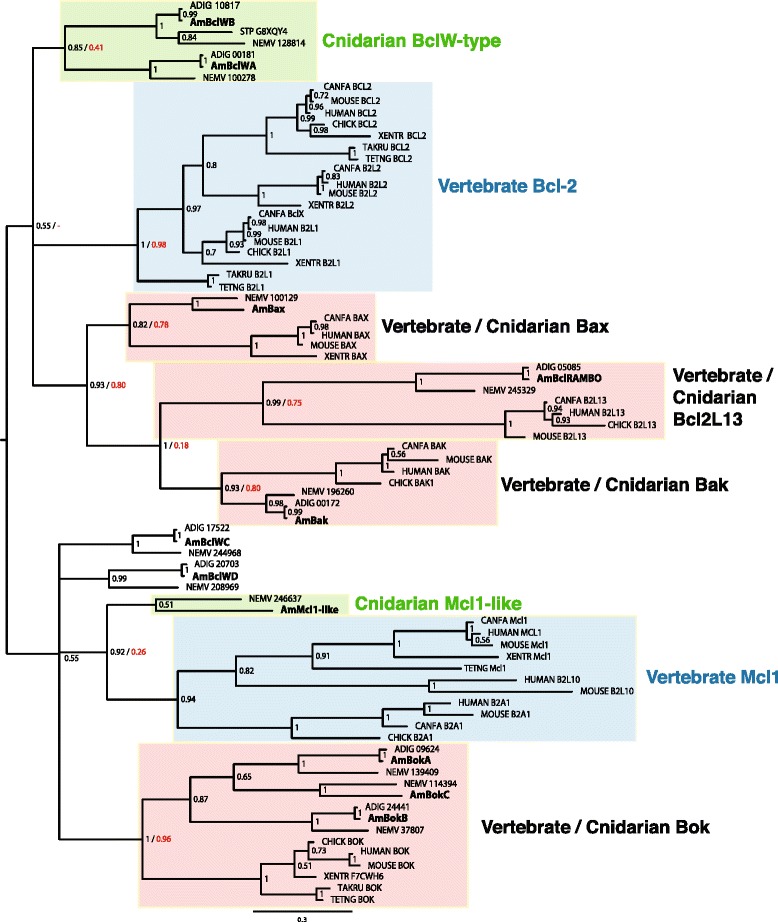
Phylogenetic analysis of Bcl-2 proteins identified in *A. millepora*. The phylogenetic tree shown was calculated using a Bayesian approach (MrBayes) based on a ClustalW alignment of Bcl-2 domains (for detailed information, see Materials and Methods). The dataset was also subjected to Maximum Likelihood analysis (Additional file 1). Posterior probability values are shown at each node, but at critical nodes, both posterior probability (*black*) and bootstrap support (*red*) data are shown as X / Y. Details of protein sequences are given as Additional file 7. Branch lengths are proportional to the number of substitutions per site in the Bayesian analysis (see scale bar in the figure). Background shading is used to emphasize relatedness of cnidarian and/or vertebrate proteins. Red backgrounds underlie well-supported clades consisting of both cnidarian and vertebrate sequences; note that the vertebrate members of these clades are exclusively pro-apoptotic. Blue or green backgrounds underlie well-supported clades consisting exclusively of vertebrate or cnidarian sequences respectively. *Acropora millepora* sequences are printed bold

Searching the *A. millepora* transcriptome [28] by a combination of HMM (Hidden Markov model, Pfam domain Bcl-2 PF00452) and BlastP analyses led to the identification of 15 candidate clusters for the Bcl-2 protein family. Transcript clusters were consolidated on the basis of sequence similarity and reference to *A. millepora* genome data (Forêt et al., unpublished data), leading us to identify 11 unique multi-domain members of the Bcl-2 family (Table 1 and Additional file 6). Each of the previously identified Bcl-2 family members was clearly represented, and similar numbers of Bcl-2 family proteins were identified in the genomes of *A. digitifera* and *N. vectensis* (see Table 1). To facilitate establishing orthology relationships, Bayesian Inference and Maximum Likelihood phylogenetic analyses were conducted on an alignment of the conserved BH domains; these results are summarized in Fig. 2 (see also Additional file 1). Note that a number of the known *Hydra* members of this family [9] are highly divergent, so the entire *Hydra* complement was excluded from this analysis. As can be seen in Fig. 2, the true vertebrate Bcl-2 proteins formed a strongly supported clade that was clearly distinct from all of the cnidarian sequences.

**Table 1:** The *Acropora millepora* repertoire of Bcl-2 family proteins

Name	Transcript ID	Best blast hit (20 Feb 2015)	*e*-value	Probable orthologs in *A. digitifera* ^*a*^	Probable orthologs in *N. vectensis* ^*a*^	BH domains	Predicted C-term TM domain	Mitochondrial association	Activity (predicted)
AmBclWA	Cluster016692	ABX61040.1 *HO088736* Bcl-like protein [*Acropora millepora*]	3e-134	ADIG 00181	NEMVE 100278	BH 1, 2, 3, 4	**+**	**+**	Anti-apoptotic
AmBclWB	Cluster004589	ABX61041.1 *DY581529* "Bax-like protein" [*Acropora millepora*]	4e-154	ADIG 10817	NEMVE 128814	BH 1, 2, 3, 4	**+**	**+**	Anti-apoptotic
AmBclWC	Cluster008158	XP_002740789.2 Apoptosis regulator R1-like [*Saccoglossus kowalevskii*]	4e-29	ADIG 17522	NEMVE 244968	BH 1, 2, 3, 4	**+**	NT	(Anti-apoptotic)
AmBclWD	Cluster011480	EKC30554.1 Bcl-2-like protein 1 [*Crassostrea gigas*]	3e-26	ADIG 20703	NEMVE 208969	BH 1, 2, 3, 4	**+**	**+**	Anti-apoptotic
AmMcl1-like	Cluster025914 Cluster034949 Cluster024735	ABA61360.1 Apoptosis regulator abhp [*Aiptasia pallida*]	2e-25	**-**	NEMVE 246637	BH 1, 2, 3, 4	**+**	**+**	Anti-apoptotic
AmBax	Cluster006261	XP_001634856.1 *EZ034459* Predicted protein [*Nematostella vectensis*]	3e-49	**-**	NEMVE 100129	BH 1, 2, 3	**+**	**+**	Pro-apoptotic strong
AmBak	Cluster009134 Cluster011792	XP_001641955.1 *EZ037140* Predicted protein [*Nematostella vectensis*]	3e-86	ADIG 00172	NEMVE 196260	BH 1, 2, 3	**+**	NT	(Pro-apoptotic)
AmBokA	Cluster022467 Cluster041039	NP_001279536.1 Bcl-2-related ovarian killer [*Callorhinchus milii*]	1e-25	ADIG 09624	NEMVE 139439	BH 1, 2, 3, 4	**-**	**+** also Golgi, ER	Anti-apoptotic weak
AmBokB	Cluster002778m	NP_001279536.1 Bcl-2-related ovarian killer [*Callorhinchus milii*]	6e-28	ADIG 24441	NEMVE 37807	BH 1, 2, 3, 4	**-**	NT	(Pro-apoptotic)
AmBokC	Cluster011056	XP_007576739.1 Bcl-2-related ovarian killer protein [*Poecilia formosa*]	2e-22	**-**	NEMVE 114394	BH 1, 2, 3	**-**	NT	(Pro-apoptotic)
AmBclRAMBO	Cluster015074	XP_001628866.1 Predicted protein [*Nematostella vectensis*]	8e-87	ADIG 05085	NEMVE 245329	BH 1, 2, 3	**+**	**+**	Pro-apoptotic

^a^see Additional file 7 for accession numbers

**Fig. 3:**
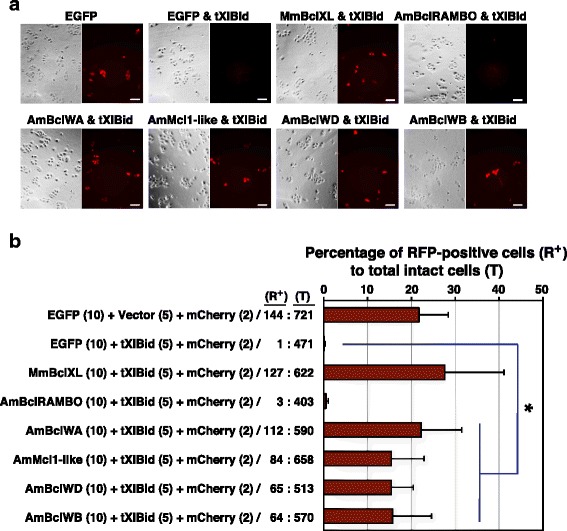
Protection assay of *A. millepora* Bcl-2 family proteins in response to extrinsic apoptotic stimuli. **a** Protection assay of coral Bcl-2 family proteins transfected in human HeLa cells against Bid-induced apoptosis. Plasmid constructs encoding *A. millepora* Bcl-2 family proteins (AmBcl-RAMBO, AmBclWA, AmMcl1-like, AmBclWD and AmBclWB), which were fused with EGFP, and an anti-apoptotic mouse Bcl-XL protein, MmBclXL, and the pCAG-mCherry plasmid were co-transfected into HeLa cells with or without pCS2-tXlBid, which encodes a potent apoptotic inducer, tXlBid. After 2 days of growth, cultures were washed to remove floating cells, and then fixed; viability was determined by monitoring mCherry-positive cells. Both phase-contrast and red fluorescence images were captured for each field under the microscope. Scale bars represent 100 μm. **b** A summary of the apoptosis protection assays. The numbers of mCherry-positive transfected and untransfected cells were counted at three distinct areas of each sample plate under the microscope, and the percentages of mCherry-positive cells to total cells were calculated. The number in parentheses indicates the ratio of the plasmid mixture. Data are presented as the means and standard deviations of samples counted from three independent experiments. An asterisk indicates *p* < 0.05 (Student’s *t*-test)

**Fig. 4:**
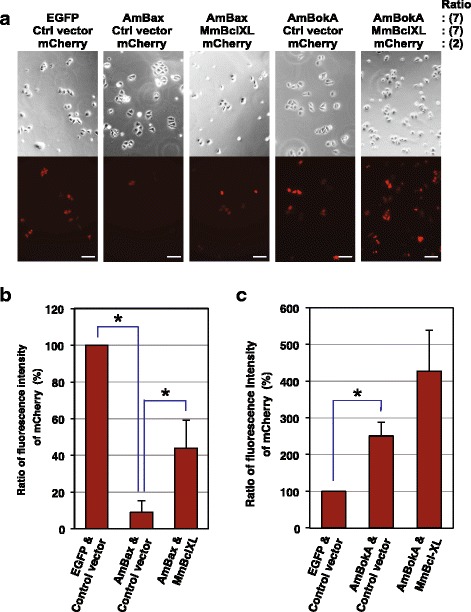
Assessment of the pro-apoptotic activity of *A. millepora* AmBax and AmBokA proteins. **a** Cytotoxicity of coral AmBax and AmBokA proteins in human HeLa cells. Plasmid constructs encoding the *A. millepora* AmBax or AmBokA proteins and the pCAG-mCherry plasmid were co-transfected into HeLa cells with (+) or without (−) pEGFP/MmBclXL. The number in parentheses indicates the ratio of the plasmid mixture. After 2 days, cultures were washed to remove floating cells and then fixed; viability was determined by monitoring mCherry-positive cells. Both phase-contrast and red fluorescence images were captured for each microscope field. Scale bars represent 100 μm. **b**, **c** Summary of cytotoxicity assays. After cultivation, transfected cells were harvested and lysed, and then fluorescence absorbance due to mCherry in each cell extract was estimated based on fluorescence intensity. Data are presented as the means and standard deviations of three independent experiments. An asterisk indicates *p* < 0.005 (Student’s *t*-test)

**Fig. 5:**
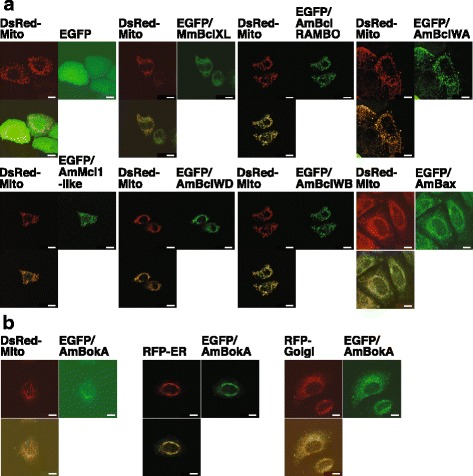
Subcellular localization of *A. millepora* Bcl-2 family proteins. **a**, **b** Plasmid constructs encoding *A. millepora* Bcl-2 family proteins fused with EGFP and plasmids encoding mCherry as a marker to visualize organelles, such as mitochondria, endoplasmic reticulum (ER) and Golgi apparatus, were transiently co-transfected. In the expression of pro-apoptotic AmBax and AmBokA, the FLAG/XlBclXL protein was co-expressed to prevent apoptosis of the transfected cells. After 2 days of cultivation, cells were washed and fixed. Both green and red fluorescent images of transfected cells were acquired using confocal microscopy and also merged into one image, resulting in an orange color in areas of co-expression. Scale bars represent 10 μm

A combination of HMM and BlastP analyses was used on the *A. millepora* transcriptome and genome and led to the identification of 11 multi-domain members of the Bcl-2 family. Each of the previously identified Bcl-2 family members was clearly represented and the corresponding GenBank accession numbers are shown in italic in third column: HO088736 [23], DY581529 [21], EZ034459, and EZ037140 [18]*.* Probable orthologs in *A. digitifera* and *N. vectensis* are shown in column 5 and 6 respectively. Scanning for the presence of a C-terminal transmembrane domain was carried out using the TMPred (http://www.ch.embnet.org/software/TMPRED_form.html) and Phobius (http://phobius.binf.ku.dk/) prediction software. Mitochondrial association was inferred from expression in mammalian cells and subsequent subcellular localization (see Results section and Fig. 5). Activity data are based on assays for pro- and anti-apoptotic assays after expression in mammalian cells (see Results section and Figs. 3, 4); parentheses indicate predicted activity where this has not yet been tested empirically. In the case of AmBclRAMBO (which is predicted to be pro-apoptotic), results were equivocal; although pro-apoptotic activity was not directly tested, no major loss of cell viability was observed upon expression of full-length protein in mammalian cells (data not shown). Note that, although the properties of AmBak were not tested, the orthologous protein from another cnidarian (*Hydra*) has pro-apoptotic activity when expressed in mammalian cells [10]. Assignments (left hand column) are based on phylogenetic analysis of Bcl-2 proteins (Fig. 2). Abbreviations: ER, endoplasmic reticulum; NT, non-tested; TM, transmembrane

A number of coral proteins clustered with specific sub-families of vertebrate Bcl-2 proteins (Bok, Bax, Bak, Bcl2L13/Bcl-RAMBO), these associations often enjoying strong bootstrap support (Fig. 2). For some other coral sequences, orthology relationships were equivocal. In the case of *A. millepora* AmMcl1-like, Bayesian analysis suggested a relationship with the vertebrate Mcl1 (Myeloid cell leukemia 1) type, but this grouping was less well supported in Maximum Likelihood (ML) analysis (Additional file 1) and, for this reason, this coral sequence is subsequently referred to as Mcl1-like. Of the vertebrate Bcl-2 proteins, four *A. millepora* sequences were most similar to the Bcl2-L2/BclW type based on alignment (Additional file 2), and hence are referred to here as AmBclWA, AmBclWB, AmBclWC and AmBclWD.

The *Acropora* AmBclWA protein (HO088736; [23]) is expressed during larval development and has also been shown to be up-regulated after 9-day exposure to 750 ppm pCO_2_ [25]. In phylogenetic analysis based on the BH domains, AmBclWA and another *A. millepora* sequence (AmBclWB- mis-identified as “Bax” by [21]) fall into a clade consisting exclusively of cnidarian sequences (Fig. 2); both of these *A. millepora* Bcl-2 proteins have clear orthologs in *A. digitifera* and *N. vectensis* (Table 1), and the latter is orthologous with G8XQY4 from *Stylophora pistillata.* Contrary to a previous report, the putative “Bax” identified in Ainsworth et al. [21] (AmBclWB; DY581529) has a full complement of BH domains and, based on its similarity to the human BclW as well as the present phylogenetic analysis (Fig. 2), is likely to be anti- rather than pro-apoptotic. This was confirmed by expression in mammalian cells (see below). On the basis of our analyses, *A. millepora* AmBax is the true coral ortholog of mammalian Bax; like the latter, it lacks a BH4 domain (Table 1). The AmBax protein was previously (correctly) identified by Pernice et al. [18] as the *Acropora* Bax ortholog (EZ034459), but this sequence was also incorrectly referred to as EU161958 in the first table of that paper. The analyses presented here also confirm that EZ037140 [18] encodes a full-length Bak-type protein (AmBak in the present study) which also lacks a BH4 domain.

Three of the *Acropora* Bcl-2 family proteins (AmBokA, AmBokB, AmBokC) cluster with the vertebrate Bok (Bcl-2-related Ovarian Killer) proteins and, like them, appear to lack the C-terminal transmembrane region typical of most Bcl-2 proteins (Table 1).

### Anti-apoptotic properties of *Acropora* Bcl-2 family members

To better understand their *in vivo* functions, coral Bcl-2 family members were expressed in mammalian cells and their ability to suppress apoptosis assessed.

In mammalian cells, the extrinsic cell-death pathway involves cleavage of the BH3-only protein Bid (Bcl-2 Interacting Domain death antagonist) by caspase-8. The resulting truncated version of the protein (tBid) triggers activation of pro-apoptotic proteins which permeabilize the outer mitochondrial membrane, releasing cytochrome *c* [29]. In human HeLa cells, it has been shown that Bid is cleaved in response to extrinsic apoptotic stimuli and its truncated form is targeted to the mitochondria [30]. The anti-apoptotic activity of Bcl-2 family members resides in their ability to prevent pro-apoptotic Bcl-2 proteins inserting into mitochondrial membranes. The ability of coral Bcl-2 family members to protect against tBid-triggered apoptosis was examined by transfecting HeLa cells simultaneously with distinct constructs encoding each Enhanced Green Fluorescent Protein (EGFP)-Bcl-2 construct (Additional file 3A), tXlBid (derived from the African clawed toad *Xenopus laevis* [31]), and mCherry (a variant of Red Fluorescent Protein RFP). Note that mCherry is required to allow visualization of intact transfected cells even at lower transfection efficiency (Additional file 3B), as the EGFP fused with coral Bcl-2 proteins was insufficient for monitoring at low magnification (data not shown). In addition, the co-expression of tX1Bid showed death inducing activity in transfected cells because no fluorescent-positive cells were observed (Additional file 3B). The expression of coral proteins in HeLa cells was confirmed by immunoblot analysis (Additional file 3C, D). As can be seen in Fig. 3a, on the basis of this assay, four of the five *A. millepora* Bcl-2 family members, including that initially identified as a (pro-apoptotic) Bax ortholog [21] (AmBclWB, lower right panel), provided comparable levels of protection relative to that conferred by mouse Bcl-XL, MmBclXL, a well-characterized anti-apoptotic Bcl-2 family member (Fig. 3b).

### Investigating the pro-apoptotic activity of *Acropora* Bax and Bok proteins

A subset of coral Bcl-2 family members (Additional file 3A) was tested for pro-apoptotic activity by expression in HeLa cells, and the results are summarized as Fig. 4. Expression of the coral Bax ortholog (AmBax) caused a large decrease in the number of viable cells (Fig. 4a, b) and this effect was partially mitigated by co-expression of anti-apoptotic mouse Bcl-XL (Fig. 4a, b). However, in this assay system the coral Bok protein (AmBokA) lacked significant pro-apoptotic activity (Fig. 4a, c). Against expectations, the data showed the enhancement of viable activity of transfected cells in the presence of AmBokA. This suggests that in cultured cells, coral AmBokA may function like human Bcl-XL, which is able to enhance transient gene expression and cell viability [32].

### Subcellular localization of *Acropora* Bcl-2 proteins

The C-terminal regions of most mammalian Bcl-2 proteins contain an α-helical transmembrane (TM) region that not only targets the proteins to the outer mitochondrial membrane, but also serves as an anchoring device. The TMPred (http://www.ch.embnet.org/software/TMPRED_form.html) and Phobius (http://phobius.binf.ku.dk/) software predicted the presence of corresponding TM motifs in most, but not all, of the coral Bcl-2 proteins (Table 1), the exceptions being the three coral Bok proteins.

For a subset of the coral Bcl-2 proteins, subcellular localization was examined after expression of EGFP-Bcl-2 fusion proteins in HeLa cells (Fig. 5); in the case of the coral Bax protein, which has strong pro-apoptotic activity (Fig. 4), subcellular localization experiments were carried out in HeLa cells that were co-expressing the (anti-apoptotic) *Xenopus* Bcl-XL protein (Fig. 5a, lower right panel). As can be seen in Fig. 5a, for each of the eight coral Bcl-2 proteins examined, fluorescence associated with the EGFP-Bcl-2 fusion protein overlapped that associated with the mitochondrial marker, DsRed-Mito, confirming association with mitochondria.

Mammalian Bok is an atypical Bcl-2 family member in that it has only a weakly hydrophobic C-terminal TM region and a significant proportion of the Bok protein is associated with membranes of the endoplasmic reticulum and Golgi apparatus [33]. As noted above, each of the three coral Bok proteins likewise either lack or have a relatively weak C-terminal TM domain, which led us to investigate whether a representative of this group (AmBokA) might be distributed like its mammalian counterparts. As can be seen in Fig. 5b, when an EGFP-coral_BokA fusion protein was overexpressed in HeLa cells, EGFP-fluorescence was associated not only with mitochondria, but also with the endoplasmic reticulum and Golgi apparatus, confirming that (at least in mammalian cells) coral BokA localizes like its mammalian counterparts.

### The *Acropora* caspase repertoire

**Fig. 6:**
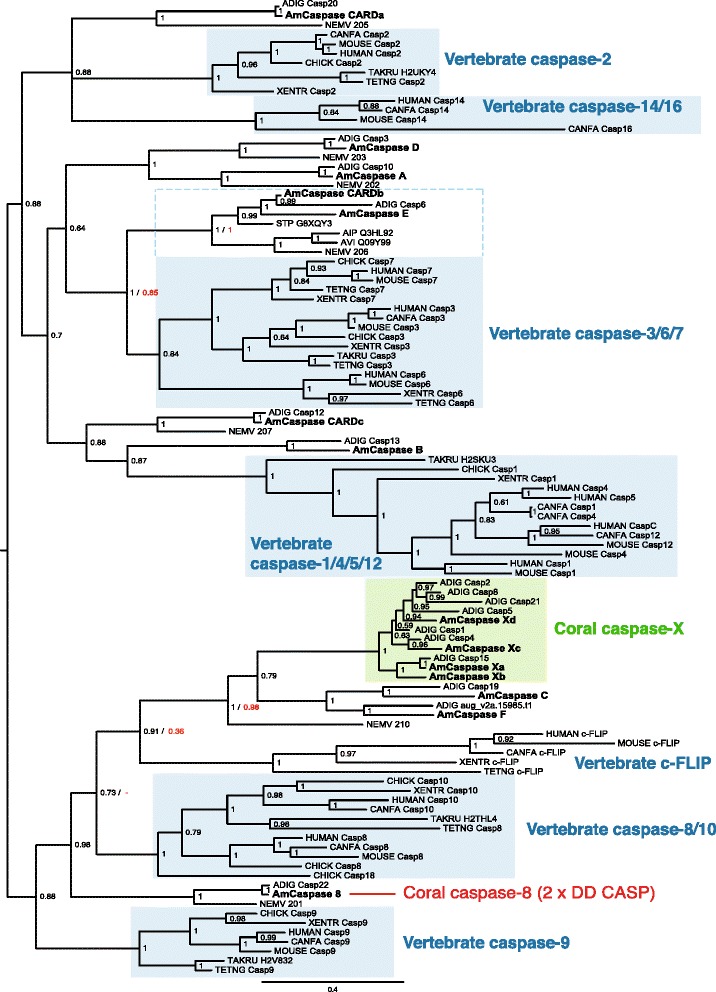
Phylogenetic analysis of caspase domains identified in *A. millepora.* The phylogeny shown was calculated using a Bayesian approach (MrBayes) based on a ClustalW alignment of Peptidase_C14 domain sequences (for detailed information, see Materials and Methods). The dataset was also subjected to Maximum Likelihood analysis (Additional file 4). Posterior probability values are shown at each node, but at critical nodes, both posterior probability (*black*) and bootstrap support (*red*) data are shown as X / Y. Note that the ML tree differs somewhat in topology (see Additional file 4), so no bootstrap figures can be attached to some nodes in the Bayesian tree shown. Details of protein sequences used in the analyses are given as Additional file 7. Branch lengths are proportional to the number of substitutions per site in the Bayesian analysis (see scale bar in the figure). Background shading is used to emphasize relatedness of cnidarian and/or vertebrate proteins. Blue backgrounds underlie well-supported clades consisting exclusively of vertebrate sequences. Green background underlies the coral caspase-X containing 2 x peptidase_C14 domains. The dashed blue line demarcates a clade of cnidarian sequences that form a well-supported sister group to the vertebrate caspase-3/6/7 clade.*Acropora millepora* sequences are printed bold

Using a combination of BlastP and HMM (Pfam domain Peptidase_C14 PF00656) search strategies, and then eliminating obvious cases of redundancy, 20 clusters likely to correspond to 14 caspase *loci* were retrieved from the *A. millepora* transcriptome (Table 2 and Additional file 6). Phylogenetic analysis of these based on caspase domain sequences (Fig. 6, Additional file 4) revealed that, whereas some of the anthozoan proteins grouped with particular vertebrate caspase classes (caspases-3, −6, −7), in many cases relationships between cnidarian sequences and the well-defined caspase types of vertebrates were equivocal.

**Table 2:** The *Acropora millepora* repertoire of caspases.

Name	Transcript ID	Best blast hit (20 Feb 2015)	*e*-value	PFAM domains	Assignment
AmCaspase A	Cluster010971	XP_008272146.1 Caspase-3 isoform X1 [*Oryctolagus cuniculus*]	9e-51	Peptidase_C14	*Caspase 3 / 6*
	Cluster011013				
	Cluster000366				
	Cluster000353				
AmCaspase B	Cluster025634	XP_006020988.1 Caspase-2 isoform X2 [*Alligator sinensis*]	1e-12	Peptidase_C14	*Caspase 1, 4, 5, 12*
AmCaspase C	No corresponding transcript cluster could be found	EKC21562.1 Caspase-8 [*Crassostrea gigas*]	1e-35	Peptidase_C14	*Caspase X-related*
AmCaspase D	Cluster003711p	XP_005972660.1 Caspase-3 [*Pantholops hodgsonii*]	2e-35	Peptidase_C14	*Caspase 3 / 6*
	Cluster004256p				
	Cluster004864p				
AmCaspase E	Cluster020870	ACH97121.1 Caspase 3-like protein [*Stylophora pistillata*]	4e-110	Peptidase_C14	*Caspases 3, 6, 7*
AmCaspase F	Cluster001637m	XP_002734388.1 Caspase-8-like isoform X1 [*Saccoglossus kowalevskii*]	7e-43	Peptidase_C14	*Caspase X-related*
AmCaspase Xa	Cluster019376	AFN02464.1 Caspase-8 [*Ciona intestinalis*]	4e-40	2 x Peptidase_C14	*Caspase X*
AmCaspase Xb	Cluster006548	ACH96579.1 Caspase-8-like cysteine peptidase [*Leucoraja erinacea*]	1e-40	2 x Peptidase_C14	*Caspase X*
AmCaspase Xc	Cluster004392	XP_009805143.1 Caspase-7 [*Gavia stellata*]	2e-34	2 x Peptidase_C14	*Caspase X*
AmCaspase Xd	Cluster007407	AFN02464.1 Caspase-8 [*Ciona intestinalis*]	2e-34	2 x Peptidase_C14	*Caspase X*
AmCaspase 8	Cluster002669p	AJG36593.1 Caspase 8 [*Acropora millepora*]	0	Peptidase_C14 2 x DED	*Caspase 8*
AmCaspase CARDa	Cluster012253	ACM46824.1 Caspase 3/9 [*Patiria pectinifera*]	1e-60	CARD Peptidase_C14	*Caspase 9*
AmCaspase CARDb	Cluster012758	ACH97121.1 Caspase 3-like protein [*Stylophora pistillata*]	7e-149	CARD Peptidase_C14	*Caspases 9*
	Cluster018373				
	Cluster005190				
AmCaspase CARDc	Cluster010805	ACM46824.1 Caspase 3/9 [*Patiria pectinifera*]	1e-65	CARD Peptidase_C14	*Caspase 1, 4, 5, 12*

A combination of HMM and BlastP analyses was used on the *A. millepora* transcriptome and genome and led to the identification of 14 members of the caspase family. Assignment (right hand column) is based on phylogenetic analysis of caspase domains (Fig. 6)

### *Acropora* caspases with N-terminal interaction domains

As indicated above, the vertebrate initiator caspases are distinguished from the effector caspases by the presence of N-terminal domains that mediate interactions with other components of the apoptotic machinery. In the Bayesian analysis (Fig. 6), *A. millepora* AmCaspase 8 and its orthologs from *A. digitifera* and *N. vectensis* are the basal group within a large clade composed of the vertebrate caspase-8, caspase-10, cellular FLICE-like inhibitory protein (c-FLIP, gene symbol *CFLAR*) and a large group of uniquely cnidarian sequences; note that vertebrate members of this clade are paralogs [34]. The AmCaspase 8 protein resembles vertebrate caspase-8 in containing two N-terminal death domains (Table 2) that can interact homotypically with the coral FADD ortholog [8]. Despite differing from bilaterian caspase-8 proteins at specific amino acid positions in the catalytic pocket, analysis of substrate specificity indicates that the coral AmCaspase 8 protein is functionally homologous with vertebrate caspase-8 [8].

Like the other vertebrate initiator caspases, several other *Acropora* caspases have N-terminal CARD (Caspase Activation and Recruitment Domain) domains (AmCaspases CARDa, CARDb and CARDc). However, molecular phylogenetics is of only limited value in predicting likely functional relationships between coral and mammal proteins (Fig. 6). For example, one of the coral CARD-caspases (AmCaspase CARDb) groups with the vertebrate effector caspases (capases-3, −6 and −7) with strong support under both Bayesian and ML analyses. Moreover, there is no consistent relationship between phylogenetics based on the caspase domain and protein domain structure – coral proteins with similar caspase domains sometimes differ with respect to presence / absence of CARD domains (e.g., AmCaspase CARDb and AmCaspase E).

### A novel class of caspase in *Acropora*

The protein type referred to here as caspase-X was first recognized in a cDNA clone (D038-A5 [23]; GenBank:KR351289). This clone encodes a protein of 721 amino acid residues that differs at a single residue (V266I) from the predicted protein product of the *A. millepora* AmCaspase Xa locus; presumably these represent alleles.

**Fig. 7:**
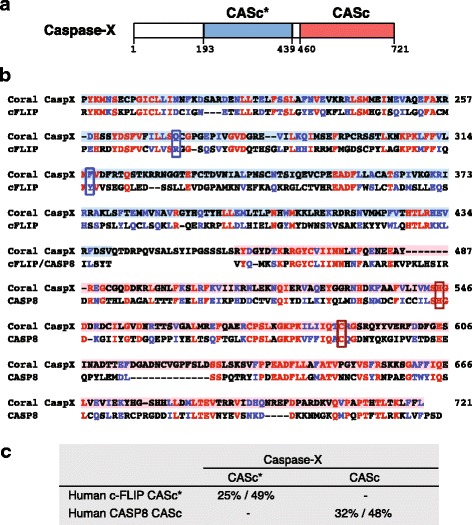
Characteristics of *A. millepora* caspase-X. **a** A schematic diagram of the *A. millepora* caspase-X protein. The pseudo protease domain, CASc*, and a protease domain, CASc, are indicated by blue and red boxes, respectively. Abbreviation: CASc, Caspase, interleukin-1 β converting enzyme homologues. **b** The alignments of amino acid sequences of CASc* and CASc motifs of *A. millepora* caspase-X with that of human c-FLIP and caspase-8 proteins. Identical and similar amino acids in the motifs are shown in red and blue, respectively. The histidine and cysteine residues outlined by red boxes are crucial amino acids for protease activity, whereas the amino acid residues outlined by blue boxes indicate the exchanged amino acids from histidine and cysteine. **c** The numbers indicate the percentage identity and similarity between two domains, respectively

Four of the *A. millepora* caspases (AmCaspases Xa, Xb, Xc, and Xd) are atypical in that they contain two caspase domains, the first of which is predicted to be inactive in each case (Fig. 7a). As can be seen in Fig. 7b and the sequence alignment shown as Additional file 5A, the second caspase domain in each of these proteins is typical in all respects (including the active site residues), whereas the first caspase-like region is atypical, lacking the catalytic Histidine and Cysteine residues, and is therefore predicted to be inactive. In phylogenetic analyses (Fig. 6), these *A. millepora* atypical caspases clustered together with seven sequences retrieved from the *A. digitifera* protein predictions. Each of these seven *A. digitifera* sequences likewise contained the basic double (inactive / active) caspase domain structure; one of the predictions (ADIG Casp4) is atypical in having four caspase domains and a glycosyl-transferase domain, and another (ADIG Casp8) also has a glycosyl-transferase domain.

The sister clade to the “double caspase” clade consists of two sequences from each of the two *Acropora* species; these are “normal” (single domain) caspases with characteristics consistent with catalytic activity.

**Fig. 8:**
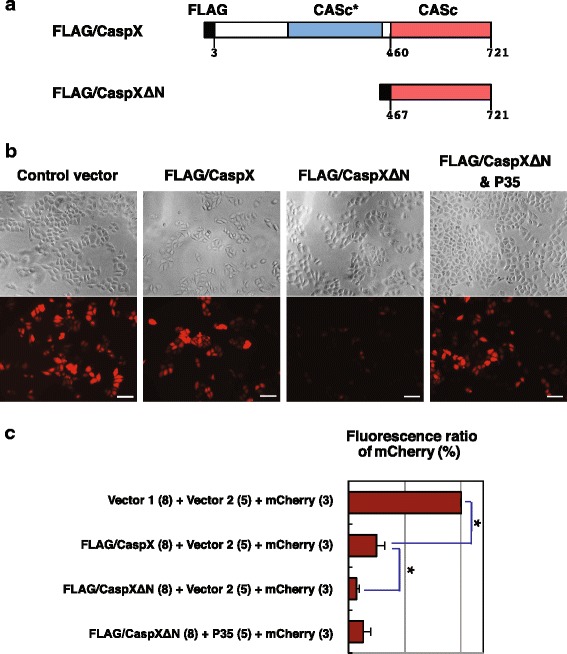
Functional activity of caspase-X. **a** A schematic diagram of constructs. FLAG/CaspX and FLAG/CaspXΔN are FLAG-tagged molecules consisting of intact or truncated caspase-X. **b** Cytotoxicity assays of human HeLa cells expressing caspase-X and its truncated forms. Plasmid constructs encoding FLAG/CaspX or FLAG/CaspXΔN and the pCAG-mCherry plasmid were co-transfected into HeLa cells in the presence or absence of baculovirus p35, which is a pan-caspase inhibitor. After 2 days of growth, cultures were washed to remove floating cells, and then fixed; viability was determined by monitoring mCherry-positive cells. Both phase-contrast and red fluorescence images were captured for each field under the microscope. Scale bars represent 100 μm. **c** A summary of cytotoxicity assays on caspase-X. After cultivation, transfected cells were harvested and lysed in the lysis buffer for the preparation of cell extracts. Fluorescence absorbance of mCherry in each cell extract was measured and viability of transfected cells was indicated by fluorescent intensity. The number in parentheses indicates the ratio of the plasmid mixture. Data are presented as the means and standard deviations of samples counted from four independent experiments (i.e. biological replicates), and are expressed relative to the empty vector (Vector 1 + Vector 2 + mCherry) value. An asterisk indicates *p* < 0.05 (Student’s *t*-test)

**Fig. 9:**
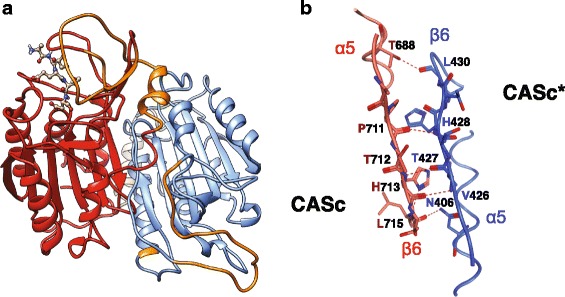
Three dimensional structural model of Caspase-X. **a** A predicted three-dimensional protein structure of caspase-X. The protease-like CASc* and protease CASc domains are shown in blue and red, respectively. The predicted disordered inter-subunit regions are shown in orange. The figure was prepared with the UCSF Chimera package [54]. **b** Closeup of the α5 helix and β6 sheet interface between CASc* (*blue*) and CASc (*red*) domains. The relative positions of amino acids involved in interactions between the two domains are shown in Additional file 5C

To examine the functional activity of caspase-X, a fusion protein, FLAG/CaspX, was generated based on cDNA clone D038-A5 (Fig. 8a; Additional file 5B), and transiently expressed in HeLa cells (Fig. 8b). A significant difference with regard to number of mCherry-positive cells was recognized in the presence and absence of FLAG/CaspX, indicating the ability of caspase-X to induce a degree of cell death. To test the idea that the first protease-like CASc* domain might regulate the activity of the second CASc domain, a truncated form, FLAG/CaspXΔN, containing only the second domain was generated (Fig. 8a; Additional file 5B), and expressed in HeLa cells (Fig. 8b) [35]. This CASc-only construct caused a further decrease in the number of mCherry-positive transfected cells, but a tendency towards recovery was observed upon co-expression of baculovirus p35 protein, which is a pan-caspase inhibitor (Fig. 8b). These cytological observations are summarized in Fig. 8c, and lead us to conclude that caspase-X has significant pro-apoptotic protease activity and that the activity of the CASc domain is negatively regulated by the presence of the CASc* domain. To better understand the relationship between the first CASc* and the second CASc domains, a three-dimensional structural model was generated (Fig. 9a). The structure consisting of the CASc* and CAS domains of caspase-X clearly resembles that of the heterocomplex of human c-FLIP CASc* domain and procaspase-8 CASc domain (Fig. 7c) [36]. As in the c-FLIP/procaspase-8 structure, the primary interface between the two domains in caspase-X is through anti-parallel association of the β6-strands in both domains. The magnified stereo view shows that several amino acids in the α5 helix and β6 sheets of the two caspase-X domains are likely to interact with each other (Fig. 9b; Additional file 5C), again as in the c-FLIP/procaspase-8 structure [36].

## Discussion

The core apoptotic machinery of *Acropora* appears to be of comparable complexity to that of other cnidarians. We reported here 11 Bcl-2 and 14 caspase family members in *A. millepora*. The Bcl-2 and caspase repertoires of the sea anemone *N. vectensis* and the freshwater hydrozoan *H. magnipapillata* have been surveyed, revealing similar levels of complexity to those reported here for *Acropora*. Nine Bcl-2 family members have been identified in *Hydra*, including two Bak-like and seven Bcl-2-like proteins [9, 10], whereas eleven family members were reported in *Nematostella*, including members of the pro-apoptotic subfamilies Bax, Bak and Bok [2]. Fifteen caspases have been reported in *Hydra*, five of which are likely to be initiators based on the presence of N-terminal DED, CARD or Death Domains (DD) [9]. The Zmasek et al. [2] survey identified 10 caspases in *Nematostella*.

Most of the multi-domain pro-apoptotic Bcl-2 family members (Bax, Bak, Bok) known from vertebrates are clearly represented in the coral and sea anemone. Consistent with conservation of function, the coral Bax ortholog (AmBax) associated with mitochondria when expressed in mammalian cells, and displayed strong pro-apoptotic activity (Fig. 4). A cnidarian Bak protein also has pro-apoptotic activity when expressed in mammalian cells [10]. However, although mammalian Bcl2L13/Bcl-RAMBO was identified as a pro-apoptotic molecule [37], overexpression of coral AmBcl-RAMBO did not induce cell death; a high proportion of cells are viable when expressing this protein fused to EGFP (data not shown), indicating that AmBcl-RAMBO does not have strong pro-apoptotic activity in mammalian cells. The mammalian Bok proteins are usually referred to as pro-apoptotic, but neither function nor mechanism is clear. The *Acropora* genome encodes at least three distinct Bok-related proteins, and in a number of respects these resemble their vertebrate counterparts. The mammalian Bok locus encodes a complete set of four BH domains (although some Bok transcripts do not), as do two (AmBokA and AmBokB) of the three coral Bok transcripts. One distinctive feature of mammalian Boks is that the C-terminal domain, which in the case of other Bcl-2 family members encodes a TM domain that serves as a tail-anchor for the mitochondrial membrane, is weakly, rather than strongly, hydrophobic. The putative coral Boks share this characteristic; for each of the three *Acropora* Bok-related proteins, both TMPred and Phobius predict either the complete absence (AmBokB and AmBokC) or the presence of only a weak (AmBokA) C-terminal TM domain. Mammalian Bok appears to be targeted to the Golgi and endoplasmic reticulum (ER) membranes in addition to the mitochondrial membrane [33], and this appears also to be the case with the coral Bok protein tested (AmBokA, Fig. 5b).

Whereas several of the coral Bcl-2 proteins appear to be orthologs of specific pro-apoptotic Bcl-2 proteins from mammals, relationships of the anti-apoptotic *Acropora* Bcl-2 proteins, including AmMcl1-like, are more ambiguous. Note that, although Bayesian analysis suggests orthology of this coral sequence with vertebrate Mcl1 proteins (Fig. 2), this grouping has limited support under ML (Additional file 1). Phylogenetics based on protease domain sequences was likewise of limited usefulness in establishing relationships between the coral and vertebrate caspase types (compare Figs. 2 and 7), and was sometimes inconsistent with predictions based on domain structure. For example, phylogenetics (Fig. 6; Additional file 4) groups AmCaspase CARDb with the vertebrate caspase-3/6/7 type (i.e. executioner caspases). However, this sequence resembles initiator caspases in encoding a CARD domain in addition to the caspase domain.

Although their phylogenetic relationships are not yet clear, the caspase-X proteins are distinguished by the presence of two predicted caspase domains, the N-terminal of which is inactive because it lacks the active site Cys and His residues (Fig. 7). The human c-FLIP protein, which likewise has an inactive protease domain, down-regulates the activity of caspase-8 for processing of most apoptotic substrates in the heterodimer complex but is able to enhance the processing of non-apoptotic substrates, such as HDAC-7 (histone deacetylase 7), by caspase-8 [38]. Although the significance of the unique domain structure of caspase-X is unknown, it may work by forming a complex with a second active domain to cleave specific substrates, as in the case of the c-FLIP and caspase-8 complex (Fig. 9B). Caspase-X proteins may be restricted to corals, as we were able to identify clearly related proteins encoded by the *A. digitifera* genome, but not by the genomes of *Nematostella* or other cnidarians. The domain structure of caspase-X proteins also appears to be unique. Searching the National Center for Biotechnology Information (NCBI) non-redundant (nr) database revealed some predicted proteins containing more than one caspase domain, often in combination with other (e.g., CARD, DD) domains, but these entries have not been verified, whereas in the case of *A. millepora* caspase-X, cDNAs have been isolated.

The present study was conducted in order to better understand the implications of a recent analysis of the responses of the coral *Acropora millepora* to CO_2_-induced ocean acidification [25]. Five distinct Bcl-2 genes were up-regulated in the longer-term (9 day) exposure, but it was unclear whether they coded for pro- or anti-apoptotic proteins. Based on the present study, 4 of the 5 up-regulated Bcl-2 s have anti-apoptotic properties, indicating that suppression of apoptosis is a key component of coral acclimation to elevated pCO_2_. The fifth Bcl-2 (Cluster015074 – AmBcl-RAMBO) is predicted to be pro-apoptotic but, as mentioned above, no major loss of viability was observed upon expression of the coral protein in mammalian cells, leaving this prediction unconfirmed.

Suppression of apoptosis (through down-regulation of the caspase-mediated apoptotic cascade) was previously proposed to be involved in coral resistance to bleaching by acting as a regulator of oxidative stress generated by symbionts [16, 17]. The juvenile corals used by Moya et al. [25] were free of symbionts but an antioxidant enzyme, catalase, was up-regulated together with Bcl-2 proteins in the longer-term pCO_2_ treatment, suggesting potential oxidative stress even in the absence of symbionts. Together, those results suggest that suppression of apoptosis might play a major role in the ability of corals to acclimate to environmental stresses.

## Conclusions

Along with the Quistad et al. [27] paper, the data presented here and elsewhere [8] indicate that the extrinsic apoptotic pathway is present in *Acropora*, whereas both *Drosophila* and *Caenorhabditis* have lost this. Moreover, although the intrinsic apoptotic pathways in the fly and worm are highly diverged, the coral system appears to be more vertebrate-like, as is the case for many other genome characteristics of cnidarians [39]. Thus the apoptotic network of the eumetazoan common ancestor was complex, and the fly and worm have derived their specialized versions from this. The extent of gene loss and divergence that has clearly occurred in the case of the apoptotic network in the model ecdysozoans has numerous precedents; for example, both the fly and worm have lost the CpG (−C-phosphate-G-) methylation system despite this being an ancestral characteristic [40].

Although there are clear functional parallels with mammals, a number of important issues remain to be resolved. Characterization of the BH3-only protein repertoire is of particular importance, but is likely to be challenging given the lack of sequence conservation typical of these molecules. The available data imply that in the coral, as in mammals, multi-domain Bcl-2 proteins modulate release of cytochrome *c* from mitochondria, allowing the assembly of the apoptosome, which also contains APAF-1 and the apoptotic initiator, caspase-9. Lange et al. [41] have previously reported the identification of the *Acropora* APAF-1 homolog [GenBank:KP170508], but the counterpart of caspase-9 is less clear – at this time, the best candidate appears to be encoded by the *A. millepora* AmCaspase CARDa, but this remains to be confirmed. Likewise, the identity of the coral death receptor (there are many candidates) and whether the signal from this complex can be amplified via the intrinsic pathway are as yet unknown. Although functional analyses are not yet possible in the coral system, *Hydra* and the sea anemone *Nematostella* - both well-established laboratory animals - are more tractable. Along with corals, these simple animals can therefore tell us a great deal more about the origins and evolution of mammalian apoptotic pathways.

## Methods

### Identification of Bcl-2 and caspase proteins

Sequences were identified by first searching the *A. millepora* transcriptome assembly [28] using a combination of HMM (Pfam domains Bcl-2 PF00452 and Peptidase_C14 PF00656) and reciprocal BlastP (e-value cut-off 1e^−5^) analyses. Proteins predicted on the basis of transcriptome data were then used to search the *A. millepora* genome (Forêt et al. unpublished data), and when possible complete coding sequences assembled by inspecting individual reads. Protein predictions used in analyses presented here are given in Additional file 6.

### Multiple sequence alignments and phylogeny reconstruction

All sequences were trimmed to one Pfam 21.0 model (Bcl-2 or Peptidase_C14 for the caspase domain [42]). Multiple sequence alignments were generated in ClustalW [43]; positions with a gap in more than 50 % of sequences were deleted. In phylogenetic analyses, MrBayes 3.2.1 was used with the following settings: 10,000,000 generations, a sample frequency of 100, a mixture of amino-acid models with fixed rate matrices and equal rates, and 25 % burn-in [44]. For the Maximum Likelihood (ML) method, the best-fitting model of protein sequence evolution was selected using ProtTest 3.2 [45] among a set of candidate models constituted through all combinations of the empirical amino acid substitution matrices. The LG + G (G = 1.957) and the WAG + G + F (G = 1.523) models were selected for the Bcl-2 and the caspase analyses respectively. PhyML 3.0 [46] starting from a random tree was used to obtain ML trees under a concatenated model assuming the amino acid substitution matrix model and the parameter values previously estimated by ProtTest 3.2. The ML support estimates were obtained after 500 (caspase tree) or 1000 (Bcl-2 tree) bootstrap replicates.

### Generation of plasmid constructs

To express coral Bcl-2 family proteins in cultured human cells, plasmid constructs were generated. Plasmids, pEGFP/AmBcl-RAMBO, pEGFP/AmBclWA, pEGFP/AmBclWB, pEGFP/AmBclWD, pEGFP/AmMcl1-like, pEGFP/AmBax, and pEGFP/AmBokA were generated by in-frame inserting the cDNA sequences, obtained by PCR amplification, into expression vector pEGFP-C1 (Invitrogen, Carlsbad, CA, USA), respectively. To express a FLAG-tagged caspase-X, the plasmid construct pCMV-FLAG/CaspX was generated by in-frame inserting a PCR-amplified caspase-X cDNA fragment into pCMV-Tag2 (Agilent Technologies, Santa Clara, CA). The DNA fragment encoding a truncated form of caspase-X, CaspXΔN, which contains the amino acid region from 467 to 721, was generated by PCR amplification, and cloned into pCMV-Tag2. To block apoptotic induction in transfected cells, three plasmid constructs were generated. A first plasmid construct, pEGFP/MmBclXL, was generated by inserting the mouse (*Mus musculus*) Bcl-XL cDNA sequence, obtained by PCR amplification, into the expression vector pEGFP-C1, and a second construct, pCAG-FLAG/XlBclXL was generated by inserting the clawed frog (*Xenopus laevis*) Bcl-XL cDNA fragment, which was obtained by PCR amplification and fused with a FLAG-tag sequence, into the expression vector pCAGGS [47]. A third plasmid construct, pCAGGS-p35, which contains the baculovirus *p35* gene encoding a pan-caspase inhibitor protein, was generated previously [48]. Generation of the plasmid construct pCAG-mCherry used for the detection of transfected cells was previously described [49]. Likewise, generation of the pCS2-tXlBid plasmid has previously been described [31]; this allows expression of tXlBid, the pro-apoptotic truncated version of the *Xenopus* Bid protein, in cultured mammalian cells. To examine the subcellular localization of coral Bcl-2 family proteins, plasmids pCS2-RFP-ER and pCS2-RFP-Golgi, were generated as previously described [50]; these and pDsRed-Mito (Clontech Laboratories Inc., Mountain View, CA, USA) were used for labeling of specific organelles.

### Cell culture and transfection

Ethics approval is not required for the present study as human cell lines were commercially obtained from ATCC (Manassas, VA, USA). Human cervical carcinoma HeLa cells and embryonic kidney HEK293T cells were cultured in Dulbecco’s Modified Eagle’s medium supplemented with 10 % fetal calf serum. Transfection of plasmid DNAs into cells was performed using Lipofectamine 2000 (Invitrogen) according to the manufacturer’s instructions.

### Assessment of anti- or pro-apoptotic activity of coral Bcl-2 family proteins and caspase-X in cultured human cells

To investigate the anti-apoptotic properties of *A. millepora* Bcl-2 family proteins, HeLa cells were transiently co-transfected with plasmids encoding coral proteins and pCAG-mCherry with or without pCS2-tXlBid. At 2 days after transfection, fragile and vulnerable cells were washed out with Phosphate Buffered Saline (PBS) and the remaining cells fixed in PBS containing 3.7 % formaldehyde. Phase-contrast and red fluorescent images were acquired with an interlined charge-coupled device (CCD) camera (CoolSNAP HQ, Roper Scientific/Photometrics, Tucson, AZ) under an inverted microscope (DMIRE2, Leica Microsystems, Wetzlar, Germany).

For the assessment of the pro-apoptotic activity of *A. millepora* AmBax and AmBokA proteins, HeLa cells were transiently co-transfected with plasmids encoding coral proteins and pCAG-mCherry with or without pEGFP-MmBclXL. To check the ability of caspase-X as an apoptotic effector, HeLa cells were transiently co-transfected with plasmids encoding intact or truncated forms of caspase-X and pCAG-mCherry with or without baculovirus p35. At 2 days after transfection, fragile and vulnerable cells were washed out with PBS and the remaining cells fixed. Phase-contrast and red fluorescent images were acquired using a fluorescence microscope. In parallel, cell extracts were prepared from transfected cells as described above, and red fluorescence intensity of cell extracts measured using the plate reader.

### Bioimaging by confocal microscopy

HeLa cells transfected with plasmid constructs were plated on a 35-mm glass-bottomed dish (Asahi Technoglass Co., Tokyo, Japan) and maintained in growth medium for 2 days. Confocal fluorescent images of cells expressing EGFP-fused coral Bcl-2 family proteins were acquired using an inverted microscope (TCS-SP5, Leica Microsystems) equipped with a 63× oil-immersion objective. Image acquisition and analyses were performed using the LAS AF Lite software system (Leica Microsystems). As mammalian Bok has been localized to the membranes of the Golgi apparatus and ER and associated membranes [33], the possibility that, when expressed in mammalian cells, the coral AmBokA protein localized to these same membranes was also examined.

### Three-dimensional modeling of *A. millepora* caspase-X

A predicted three-dimensional model of the protein structure of caspase-X was built based on alignment with 3H11 [36], which is the heterodimeric complex of c-FLIP and procaspase-8, using the Molecular Operating Environment (MOE) software package (MOE version 2013.08, Chemical Computing Group Inc., Tokyo). The disordered regions of the coral caspase-X protein were predicted by DISOPRED3 [51].

## Availability of supporting data

Bcl-2 and caspase protein predictions used in analyses presented here are given in Additional file 6.

Phylogenetic data (alignments and phylogenetic trees) have been deposited to TreeBase and are accessible via the URL: http://purl.org/phylo/treebase/phylows/study/TB2:S18551).

## Additional files

Additional file 1:
**Phylogenetic analysis of Bcl-2 proteins identifed in** ***A. millepora*** **inferred by the Maximum Likelihood method.** PhyML 3.0 starting from a random tree was used to obtain ML trees under a concatenated model assuming the LG + G (G = 1.957) amino acid substitution matrix model. The ML bootstraps proportion was obtained after 1000 bootstrap replicates. (PDF 267 kb) Additional file 2:
**Alignment of** ***A. millepora*** **AmBclWA and AmBclWD with Human Bcl-W.** Red indicates identical amino acids, blue similar amino acids. (PDF 236 kb) Additional file 3:
**Plasmid constructs and immunoblot analyses of** ***A. millepora*** **Bcl-2 family proteins.** (**A**) A schematic diagram of the EGFP-fused *A. millepora* Bcl-2 family proteins. Figures in parentheses are the number of amino acid residues in each construct. (**B**) Microscopic observation of transfected cells expressing EGFP and mCherry together with or without tX1Bid. Plasmid constructs pEGFP-C1 and pCAG-mCherry were co-transfected into HeLa cells either with pCS2-tX1Bid or control pCS2 empty vector. After 2 days of growth, cultures were washed to remove floating cells, and then fixed. The phase-contrast and both green and red fluorescent images of the remaining attached cells were captured for each field under the microscope. Scale bars represent 100 μm. (**C, D**) Immunoblot analysis of fusion proteins. Plasmids encoding each fusion protein were transiently transfected into HEK293T cells. In the case of EGFP/AmBax and EGFP/AmBokA proteins, the plasmid construct pCAG-FLAG/XlBclXL was cotransfected to prevent cell death. After culture for 2 days, transgene products were analyzed with control cell extracts by immunoblotting with appropriate antibodies. Arrowheads indicate the positions of immunoreactive proteins. Additional details of reagents and immunoblot analysis are provided as Additional file 8. Abbreviation: MWM, molecular weight marker. (PDF 2361 kb) Additional file 4:
**Phylogenetic analysis of caspase domains identifed in** ***A. millepora*** **inferred by Maximum Likelihood method.** PhyML 3.0 starting from a random tree was used to obtain ML trees under a concatenated model assuming the WAG + G + F (G = 1.523) amino acid substitution matrix model. The ML bootstrap proportion was obtained after 500 bootstraps replicates. Background colouring is as in Fig. 6. (PDF 361 kb) Additional file 5:
**Similarity of caspase-X paralogs and immunoblot analysis of** ***A. millepora*** **caspase-X constructs.** (**A**) Alignment of the caspase-X paralogs from *A. millepora*. The red underlined section is a typical caspase domain, with the catalytic residues (H and C) highlighted. The blue underlined section is an additional caspase-like domain, but lacking the catalytic residues (highlight indicates positions where the catalytic residues should be, based on the alignment). Numbering at left refers to residue position in the caspase-X sequence; note that, in the region shown, the caspase-X sequence (derived from cDNA clone D038-A5, GenBank:KR351289) differs at only one position (V266I) from the genomic prediction AmCaspase Xa. (**B**) Immunoblot analysis of FLAG/CaspX and its truncated form. Plasmids encoding intact or truncated forms of caspase-X with a FLAG-tag were transiently cotransfected into HEK293T cells together with the pCAG-p35 plasmid. After culture for 2 days, transgene products were analyzed with control cell extracts by immunoblotting with appropriate antibodies. The asterisk indicates a non-specific reaction. Abbreviation: MWM, molecular weight marker. (**C**) The assignments of α-helix and β-sheet secondary structure elements are based on a previous study [52]. Residues predicted to form H bonds between the two domains are indicated by asterisks. (PDF 1084 kb) Additional file 6:
**Bcl-2 and caspase protein predictions for the coral** ***A. millepora.*** (XLS 23 kb) Additional file 7:
**Accession numbers of sequences used for the Bcl-2 and caspase phylogenies.** (XLS 51 kb) Additional file 8:
**Additional information regarding reagents and immunoblot analysis.** (DOC 82 kb)

## Abbreviations

APAF-1: Apoptotic Peptidase-Activating Factor 1
BAK: Bcl-2 Antagonist Killer 1
BAX: Bcl-2-Associated X protein
BH: Bcl-2 Homology
BID: Bcl-2 Interacting Domain death antagonist
BOK: Bcl-2-related Ovarian Killer
c-FLIP: cellular FLICE-Like Inhibitory Protein
CARD: Caspase Activation and Recruitment Domain
Caspase: Cysteine-dependent Aspartyl-Specific Protease
Cyt-*c*: Cytochrome *c*
DD: Death Domain
DED: Death Effector Domain
EGFP: Enhanced Green Fluorescent Protein
ER: Endoplasmic Reticulum
FADD: Fas-Associated protein with Death Domain
HMM: Hidden Markov Model
Mcl1: Myeloid Cell Leukemia 1
ML: Maximum Likelihood
PBS: Phosphate Buffered Saline
TM: TransMembrane

## References

[1] Sakamaki K, Imai K, Tomii K, Miller D. Evolutionary analyses of caspase-8 and its paralogs: deep origins of the apoptotic signaling pathways. Bioessays. in press. doi:10.1002/bies.201500010. 10.1002/bies.201500010 26010168

[2] Zmasek C, Zhang Q, Ye Y, Godzik A (2007). Surprising complexity of the ancestral apoptosis network. Genome Biol.

[3] Jacobson M, Weil M, Raff M (1997). Programmed cell death in animal development. Cell.

[4] Srinivasula S, Saleh A, Ahmad M, Fernandes-Alnemri T, Alnemri E (2001). Isolation and assay of caspases. Methods Cell Biol.

[5] Oberst A, Bender C, Green D (2008). Living with death: the evolution of the mitochondrial pathway of apoptosis in animals. Cell Death Differ.

[6] Czabotar P, Lessene G, Strasser A, Adams J (2014). Control of apoptosis by the BCL-2 protein family: implications for physiology and therapy. Nat Rev Mol Cell Biol.

[7] Kornbluth S, White K (2005). Apoptosis in *Drosophila*: neither fish nor fowl (nor man, nor worm). J Cell Sci.

[8] Sakamaki K, Shimizu K, Iwata H, Imai K, Satou Y, Funayama N (2014). The apoptotic initiator caspase-8: its functional ubiquity and genetic diversity during animal evolution. Mol Biol Evol.

[9] Lasi M, David C, Böttger A (2010). Apoptosis in pre-Bilaterians: *Hydra* as a model. Apoptosis.

[10] Lasi M, Pauly B, Schmidt N, Cikala M, Stiening B, Käsbauer T (2010). The molecular cell death machinery in the simple cnidarian *Hydra* includes an expanded caspase family and pro-and anti-apoptotic Bcl-2 proteins. Cell Res.

[11] Seipp S, Schmich J, Leitz T (2001). Apoptosis–a death-inducing mechanism tightly linked with morphogenesis in *Hydractina echinata* (Cnidaria, Hydrozoa). Development.

[12] Seipp S, Wittig K, Stiening B, Bottger A, Leitz T (2006). Metamorphosis of *Hydractinia echinata* (Cnidaria) is caspase-dependent. Int J Dev Biol.

[13] Wittig K, Kasper J, Seipp S, Leitz T (2011). Evidence for an instructive role of apoptosis during the metamorphosis of *Hydractinia echinata* (Hydrozoa). Zoology.

[14] Lesser MP, Dubinsky Z, Stambler N (2011). Coral bleaching: causes and mechanisms. Coral Reefs: An Ecosystem in Transition.

[15] Tolleter D, Seneca F, DeNofrio J, Krediet C, Palumbi S, Pringle J (2013). Coral bleaching independent of photosynthetic activity. Curr Biol.

[16] Tchernov D, Kvitt H, Haramaty L, Bibby T, Gorbunov M, Rosenfeld H (2011). Apoptosis and the selective survival of host animals following thermal bleaching in zooxanthellate corals. Proc Natl Acad Sci U S A.

[17] Kvitt H, Rosenfeld H, Zandbank K, Tchernov D (2011). Regulation of apoptotic pathways by *Stylophora pistillata* (Anthozoa, Pocilloporidae) to survive thermal stress and bleaching. PLoS One.

[18] Pernice M, Dunn S, Miard T, Dufour S, Dove S, Hoegh-Guldberg O (2011). Regulation of apoptotic mediators reveals dynamic responses to thermal stress in the reef building coral *Acropora millepora*. PLoS One.

[19] Dunn S, Phillips W, Spatafora J, Green D, Weis V (2006). Highly conserved caspase and Bcl-2 homologues from the sea anemone *Aiptasia pallida*: lower metazoans as models for the study of apoptosis evolution. J Mol Evol.

[20] Zmasek C, Godzik A (2013). Evolution of the animal apoptosis network. Cold Spring Harb Perspect Biol.

[21] Ainsworth T, Hoegh-Guldberg O, Heron S, Skirving W, Leggat W (2008). Early cellular changes are indicators of pre-bleaching thermal stress in the coral host. J Exp Mar Biol Ecol.

[22] Bellantuono A, Granados-Cifuentes C, Miller D, Hoegh-Guldberg O, Rodriguez-Lanetty M (2012). Coral thermal tolerance: tuning gene expression to resist thermal stress. PLoS One.

[23] Grasso L, Negri A, Forêt S, Saint R, Hayward D, Miller D (2011). The biology of coral metamorphosis: molecular responses of larvae to inducers of settlement and metamorphosis. Dev Biol.

[24] Kaniewska P, Campbell P, Kline D, Rodriguez-Lanetty M, Miller D, Dove S (2012). Major cellular and physiological impacts of ocean acidification on a reef building coral. PLoS One.

[25] Moya A, Huisman L, Forêt S, Gattuso J, Hayward D, Ball E (2015). Rapid acclimation of juvenile corals to CO2-mediated acidification by up-regulation of HSP and Bcl-2 genes. Mol Ecol.

[26] Shinzato C, Shoguchi E, Kawashima T, Hamada M, Hisata K, Tanaka M (2011). Using the *Acropora digitifera* genome to understand coral responses to environmental change. Nature.

[27] Quistad S, Stotland A, Barott K, Smurthwaite C, Hilton B, Grasis J (2014). Evolution of TNF-induced apoptosis reveals 550 My of functional conservation. Proc Natl Acad Sci U S A.

[28] Moya A, Huisman L, Ball E, Hayward D, Grasso L, Chua C (2012). Whole transcriptome analysis of the coral *Acropora millepora* reveals complex responses to CO2-driven acidification during the initiation of calcification. Mol Ecol.

[29] Li H, Zhu H, Xu C, Yuan J (1998). Cleavage of BID by caspase 8 mediates the mitochondrial damage in the Fas pathway of apoptosis. Cell.

[30] Schug Z, Gonzalvez F, Houtkooper R, Vaz F, Gottlieb E (2011). BID is cleaved by caspase-8 within a native complex on the mitochondrial membrane. Cell Death Differ.

[31] Kominami K, Takagi C, Kurata T, Kitayama A, Nozaki M, Sawasaki T (2006). The initiator caspase, caspase-10b, and the BH-3-only molecule, Bid, demonstrate evolutionary conservation in *Xenopus* of their pro-apoptotic activities in the extrinsic and intrinsic pathways. Genes Cells.

[32] Majors B, Betenbaugh M, Pederson N, Chiang G (2008). Enhancement of transient gene expression and culture viability using Chinese hamster ovary cells overexpressing Bcl-x(L). Biotechnol Bioeng.

[33] Echeverry N, Bachmann D, Ke F, Strasser A, Simon H, Kaufmann T (2013). Intracellular localization of the BCL-2 family member BOK and functional implications. Cell Death Differ.

[34] Sakamaki K, Satou Y (2009). Caspases: evolutionary aspects of their functions in vertebrates. J Fish Biol.

[35] Zhou Q, Krebs J, Snipas S, Price A, Alnemri E, Tomaselli K (1998). Interaction of the baculovirus anti-apoptotic protein p35 with caspases. Specificity, kinetics, and characterization of the caspase/p35 complex. Biochemistry.

[36] Yu J, Jeffrey P, Shi Y (2009). Mechanism of procaspase-8 activation by c-FLIPL. Proc Natl Acad Sci U S A.

[37] Kataoka T, Holler N, Micheau O, Martinon F, Tinel A, Hofmann K (2001). Bcl-rambo, a novel Bcl-2 homologue that induces apoptosis via its unique C-terminal extension. J Biol Chem.

[38] Pop C, Oberst A, Drag M, Van Raam B, Riedl S, Green D (2011). FLIP(L) induces caspase 8 activity in the absence of interdomain caspase 8 cleavage and alters substrate specificity. Biochem J.

[39] Miller D, Ball E (2008). Cryptic complexity captured: the *Nematostella* genome reveals its secrets. Trends Genet.

[40] Lyko F, Maleszka R (2011). Insects as innovative models for functional studies of DNA methylation. Trends Genet.

[41] Lange C, Hemmrich G, Klostermeier U, López-Quintero J, Miller D, Rahn T (2011). Defining the origins of the NOD-like receptor system at the base of animal evolution. Mol Biol Evol.

[42] Bateman A, Coin L, Durbin R, Finn R, Hollich V, Griffiths-Jones S (2004). The Pfam protein families database. Nucleic Acids Res.

[43] Larkin M, Blackshields G, Brown N, Chenna R, McGettigan P, McWilliam H (2007). Clustal W and Clustal X version 2.0.. Bioinformatics.

[44] Ronquist F, Huelsenbeck J (2003). MrBayes 3: Bayesian phylogenetic inference under mixed models. Bioinformatics.

[45] Abascal F, Zardoya R, Posada D (2005). ProtTest: Selection of best-fit models of protein evolution. Bioinformatics.

[46] Guindon S, Dufayard J, Lefort V, Anisimova M, Hordijk W, Gascuel O (2010). New algorithms and methods to estimate Maximum-Likelihood phylogenies: assessing the performance of PhyML 3.0.. Syst Biol.

[47] Niwa H, Yamamura K, Miyazaki J (1991). Efficient selection for high-expression transfectants with a novel eukaryotic vector. Gene.

[48] Sakamaki K, Takagi C, Kominami K, Sakata S, Yaoita Y, Kubota H (2004). The adaptor molecule FADD from *Xenopus laevis* demonstrates evolutionary conservation of its pro-apoptotic activity. Genes Cells.

[49] Sakamaki K, Takagi C, Kitayama A, Kurata T, Yamamoto T, Chiba K (2012). Multiple functions of FADD in apoptosis, NF-kappaB-related signaling, and heart development in *Xenopus* embryos. Genes Cells.

[50] Takagi C, Sakamaki K, Morita H, Hara Y, Suzuki M, Kinoshita N (2013). Transgenic *Xenopus laevis* for live imaging in cell and developmental biology. Dev Growth Differ.

[51] Jones D, Cozzetto D (2014). DISOPRED3: precise disordered region predictions with annotated protein-binding activity. Bioinformatics.

[52] Watt W, Koeplinger K, Mildner A, Heinrikson R, Tomasselli A, Watenpaugh K (1999). The atomic-resolution structure of human caspase-8, a key activator of apoptosis. Structure.

[53] Moldoveanu T, Folis A, Kriwacki R, Green D (2014). Many players in BCL-2 family affairs. Trends Biochem Sci.

[54] Pettersen E, Goddard T, Huang C, Couch G, Greenblatt D, Meng E (2004). UCSF Chimera – a visualization system for exploratory research and analysis. J Comput Chem.

