# Supplementary figures and images for: Functional conservation of the apoptotic machinery from coral to man: the diverse and complex Bcl-2 and caspase repertoires of Acropora millepora
Source: BMC Genomics. 2016 Jan 16;17:62. doi: 10.1186/s12864-015-2355-x (PMC4715348; doi:10.1186/s12864-015-2355-x)

Additional file 1

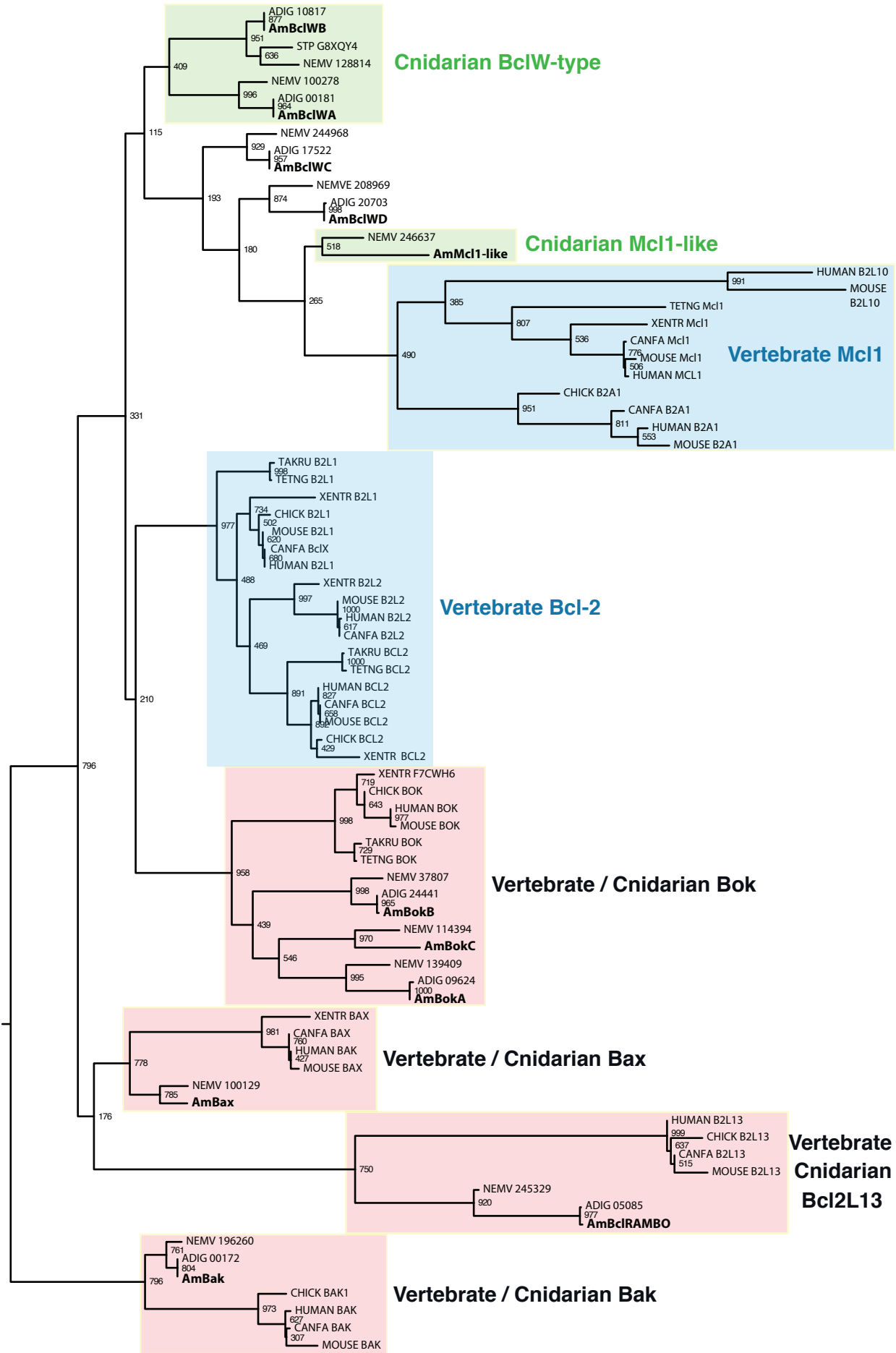

Supplement: Additional file 1: — Phylogenetic analysis of Bcl-2 proteins identifed in A. millepora inferred by the Maximum Likelihood method. PhyML 3.0 starting from a random tree was used to obtain ML trees under a concatenated model assuming the LG + G (G = 1.957) amino acid substitution matrix model. The ML bootstraps proportion was obtained after 1000 bootstrap replicates. (PDF 267 kb) [file 12864_2015_2355_MOESM1_ESM.pdf]

# Additional file 3

A

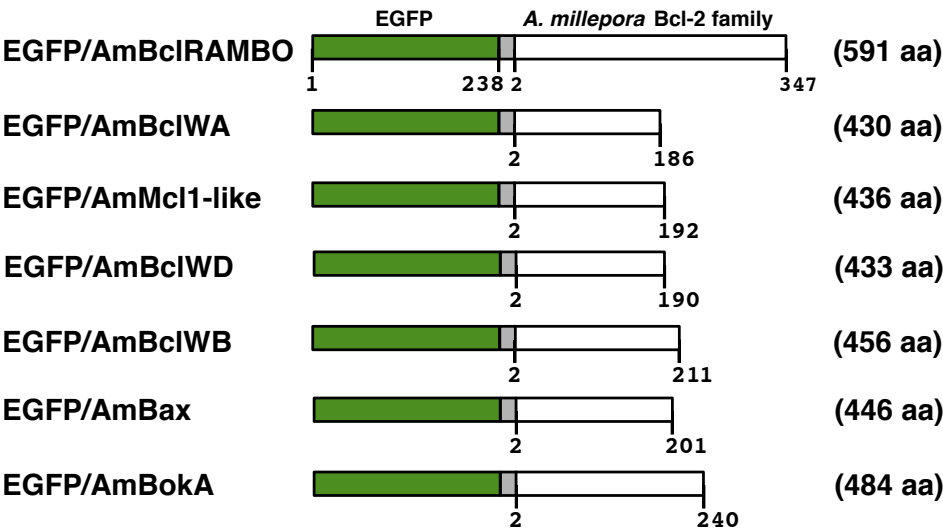

B

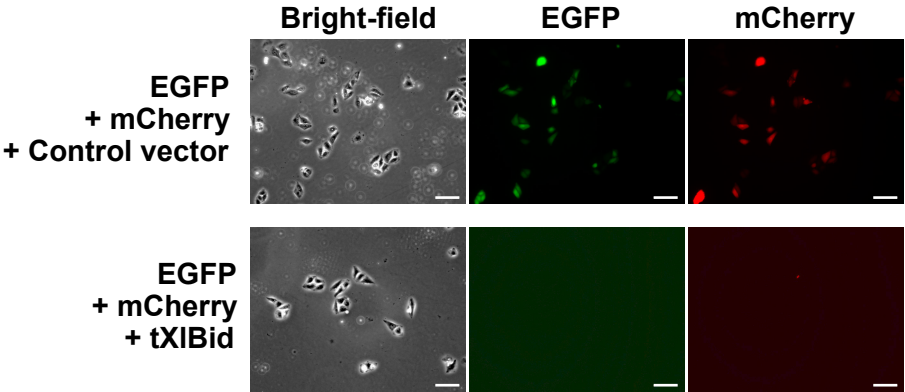

C

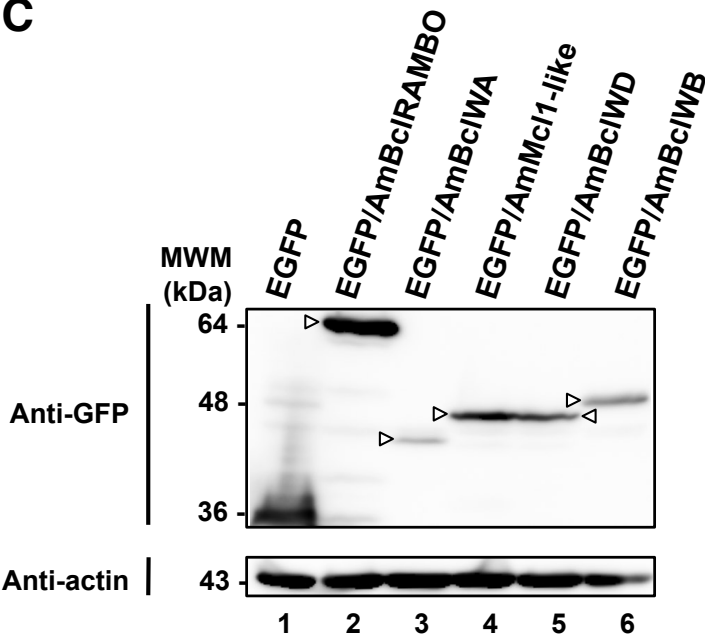

D

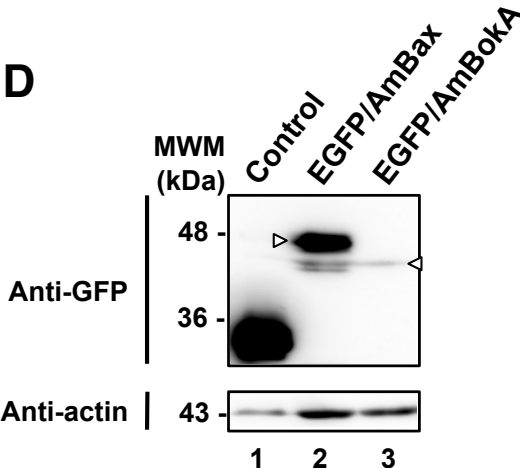

Supplement: Additional file 3: — Plasmid constructs and immunoblot analyses of A. millepora Bcl-2 family proteins. (A) A schematic diagram of the EGFP-fused A. millepora Bcl-2 family proteins. Figures in parentheses are the number of amino acid residues in each construct. (B) Microscopic observation of transfected cells expressing EGFP and mCherry together with or without tX1Bid. Plasmid constructs pEGFP-C1 and pCAG-mCherry were co-transfected into HeLa cells either with pCS2-tX1Bid or control pCS2 empty vector. After 2 days of growth, cultures were washed to remove floating cells, and then fixed. The phase-contrast and both green and red fluorescent images of the remaining attached cells were captured for each field under the microscope. Scale bars represent 100 μm. (C, D) Immunoblot analysis of fusion proteins. Plasmids encoding each fusion protein were transiently transfected into HEK293T cells. In the case of EGFP/AmBax and EGFP/AmBokA proteins, the plasmid construct pCAG-FLAG/XlBclXL was cotransfected to prevent cell death. After culture for 2 days, transgene products were analyzed with control cell extracts by immunoblotting with appropriate antibodies. Arrowheads indicate the positions of immunoreactive proteins. Additional details of reagents and immunoblot analysis are provided as Additional file 8. Abbreviation: MWM, molecular weight marker. (PDF 2361 kb) [file 12864_2015_2355_MOESM3_ESM.pdf]

## Additional file 4

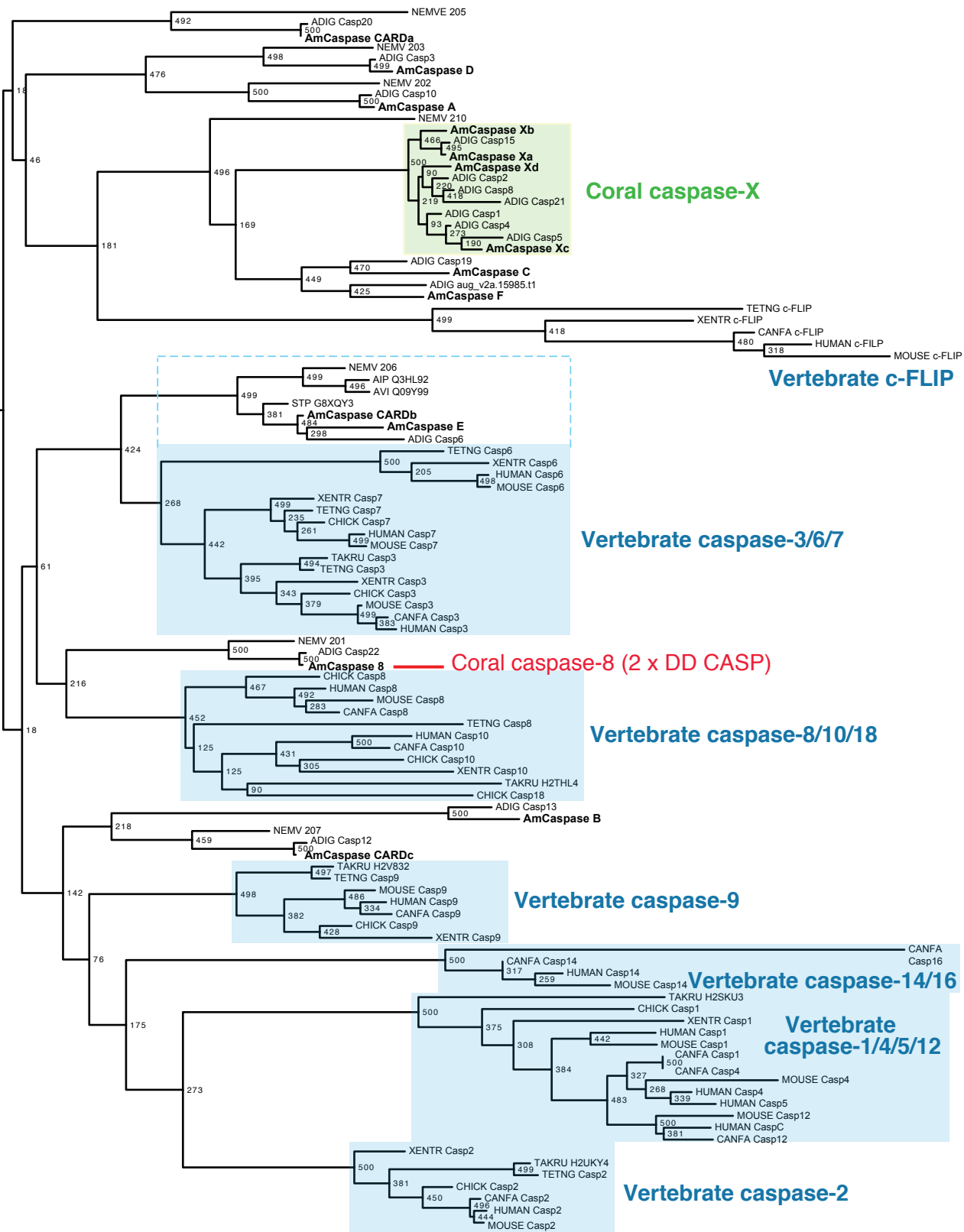

Supplement: Additional file 4: — Phylogenetic analysis of caspase domains identifed in A. millepora inferred by Maximum Likelihood method. PhyML 3.0 starting from a random tree was used to obtain ML trees under a concatenated model assuming the WAG + G + F (G = 1.523) amino acid substitution matrix model. The ML bootstrap proportion was obtained after 500 bootstraps replicates. Background colouring is as in Fig. 6. (PDF 361 kb) [file 12864_2015_2355_MOESM4_ESM.pdf]
