# Supplementary material for: Functional conservation of the apoptotic machinery from coral to man: the diverse and complex Bcl-2 and caspase repertoires of Acropora millepora
Source: BMC Genomics. 2016 Jan 16;17:62. doi: 10.1186/s12864-015-2355-x (PMC4715348; doi:10.1186/s12864-015-2355-x)
Supplement: Additional file 2: — Alignment of A. millepora AmBclWA and AmBclWD with Human Bcl-W. Red indicates identical amino acids, blue similar amino acids. (PDF 236 kb) [file 12864_2015_2355_MOESM2_ESM.pdf]

Additional file 2

```
Coral BclWA     MVHNNDRLRSFNTIVKDYVGYKLRQKNIFLDGYNVNES--PSAAACH--LRRVADELIE
Coral BclWD   MNCIQSEKIAGPANEEMLRIGEKARSLARDLISYRVGSVNTPPPSRTALILRRLADGLED
Human BclW        MATPASAPDTRALVADFVGYKLRQK-GYVCGAGPGEG--PAADPLHQAMRAAGDEFET
                                 BH4                                BH3

Coral BclWA   ENRQLFDSMCDQLHLTHASTYATFVGIADEIFQTGKNWGRIVAFLAFGATLAVYCVQKED
Coral BclWD   SHSVVLANMCNRLNVLSGTARSKFVQVADEVFRDGINWGRIVAVYAFGAKLSQYCMR-NG
Human BclW    RFRRTFSDLAAQLHVTPGSAQQRFTQVSDELFQGGPNWGRLVAFFVFGAALCAESVN-KE
                                                   BH1

Coral BclWA   LAELLDNIIEWLSLYMEQNLGQWINENGGWEGFIQFFKK----EDGSPGNGRNGGWRIAA
Coral BclWD   LEDDVAEVVLWLGNYISG-LSAWIQAQGGWPSFDKTFGD-------ALEEREKVWWKKIC
Human BclW    MEPLVGQVQEWMVAYLETQLADWIHSSGGWAEFTALYGDGALEEARRLREGNWASVRTVL
                                   BH2                                 TM

Coral Bcl2WA  VAGLGIGALLMLACR
Coral Bcl2WD  LAAVGFGAIATLIYQQSTA
Human BclW    TGAVALGALVTVGAFFASK
```
