# Supplementary material for: Functional conservation of the apoptotic machinery from coral to man: the diverse and complex Bcl-2 and caspase repertoires of Acropora millepora
Source: BMC Genomics. 2016 Jan 16;17:62. doi: 10.1186/s12864-015-2355-x (PMC4715348; doi:10.1186/s12864-015-2355-x)

Additional file 5

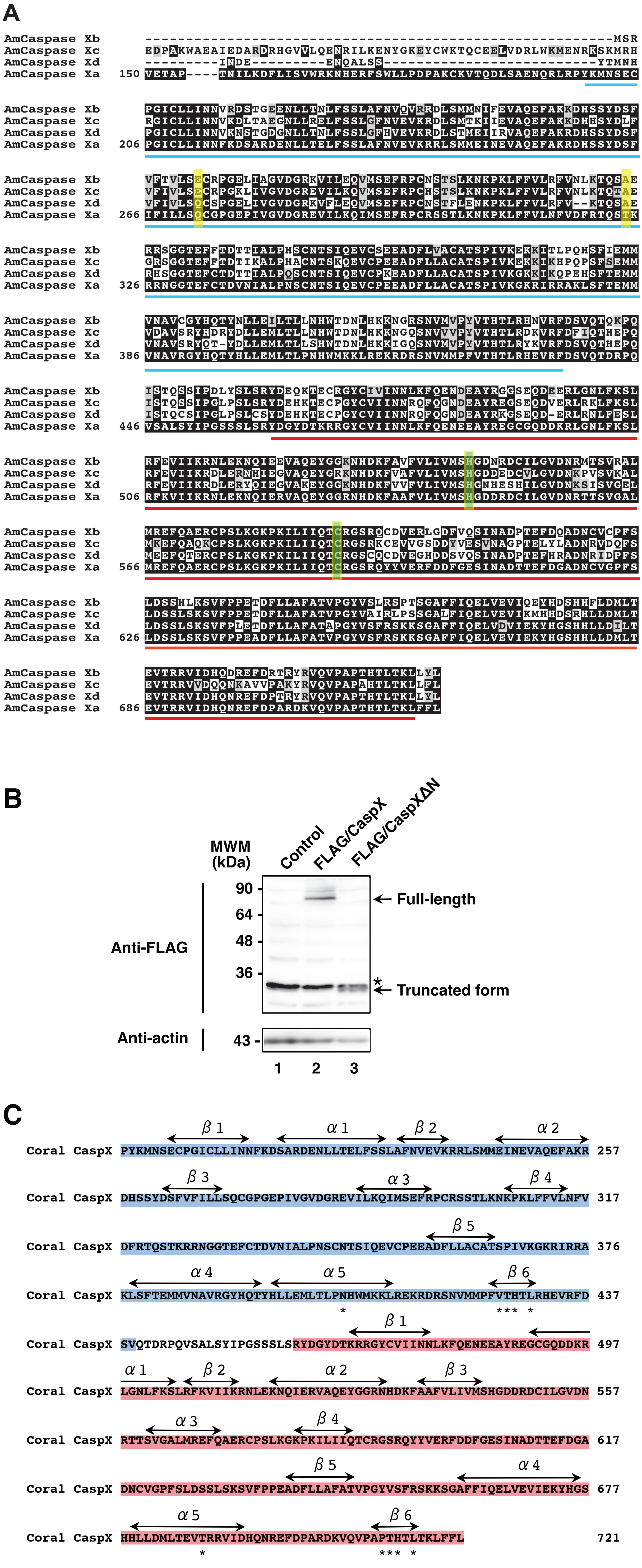

Supplement: Additional file 5: — Similarity of caspase-X paralogs and immunoblot analysis of A. millepora caspase-X constructs. (A) Alignment of the caspase-X paralogs from A. millepora. The red underlined section is a typical caspase domain, with the catalytic residues (H and C) highlighted. The blue underlined section is an additional caspase-like domain, but lacking the catalytic residues (highlight indicates positions where the catalytic residues should be, based on the alignment). Numbering at left refers to residue position in the caspase-X sequence; note that, in the region shown, the caspase-X sequence (derived from cDNA clone D038-A5, GenBank:KR351289) differs at only one position (V266I) from the genomic prediction AmCaspase Xa. (B) Immunoblot analysis of FLAG/CaspX and its truncated form. Plasmids encoding intact or truncated forms of caspase-X with a FLAG-tag were transiently cotransfected into HEK293T cells together with the pCAG-p35 plasmid. After culture for 2 days, transgene products were analyzed with control cell extracts by immunoblotting with appropriate antibodies. The asterisk indicates a non-specific reaction. Abbreviation: MWM, molecular weight marker. (C) The assignments of α-helix and β-sheet secondary structure elements are based on a previous study [52]. Residues predicted to form H bonds between the two domains are indicated by asterisks. (PDF 1084 kb) [file 12864_2015_2355_MOESM5_ESM.pdf]
