# Supplementary material for: Functional conservation of the apoptotic machinery from coral to man: the diverse and complex Bcl-2 and caspase repertoires of Acropora millepora
Source: BMC Genomics. 2016 Jan 16;17:62. doi: 10.1186/s12864-015-2355-x (PMC4715348; doi:10.1186/s12864-015-2355-x)
Supplement: Additional file 8: — Additional information regarding reagents and immunoblot analysis. (DOC 82 kb) [file 12864_2015_2355_MOESM8_ESM.doc]

**Additional file 8**

**Reagents:** Antibodies were obtained from the indicated suppliers: anti-GFP (GF200, Nacalai Tesque), anti-FLAG (M2, Sigma-Aldrich, St. Louis, MO, USA), anti-actin (MAB1501R, Chemicon International Inc., Temecula, CA, USA), and HRP-conjugated anti-mouse IgG antibody (Cell Signaling Technology, Danvers, MA, USA).

**Immunoblot analysis:** To detect ectopic expression of coral Bcl-2 family proteins or caspase-X protein in cultured mammalian cells, plasmids encoding proteins fused with EGFP or FLAG-tag were transiently transfected into HEK293T cells.

In the case of the detection of AmBax, AmBokA, and caspase-X proteins, the plasmid construct pCAG-FLAG/XlBclXL or pCAG-p35 was cotransfected into HEK293T cells to prevent cell death. After 2 days of cultivation, transfected cells were lysed in lysis buffer [50 mM Tris-HCl (pH 7.5), 20 mM MgCl_2_, 0.5% Nonidet P-40, 150 mM NaCl and protease inhibitor cocktails (Nacalai Tesque)]. After cell debris was removed by centrifugation, all cell lysates were denatured in the Laemmli sample buffer, resolved by SDS-PAGE, and analyzed by immunoblotting with anti-GFP, anti-FLAG, and anti-actin antibodies. After incubation with an HRP-conjugated anti-mouse IgG antibody, immune complexes were visualized with Immobilon^TM^ Western (Millipore Corporation, Billerica, MA, USA) using a luminescent image analyzer (LAS-3000, Fujifilm, Tokyo, Japan).
